# Mifepristone, preemption, and public health federalism

**DOI:** 10.1093/jlb/lsac037

**Published:** 2022-12-21

**Authors:** Patricia J Zettler, Annamarie Beckmeyer, Beatrice L Brown, Ameet Sarpatwari

**Affiliations:** Moritz College of Law, Drug Enforcement and Policy Center, Cancer Control Program, The James Comprehensive Cancer Center, The Ohio State University, Columbus, OH, USA; College of Medicine, The Ohio State University, Columbus, OH, USA; Program On Regulation, Therapeutics, And Law (PORTAL), Division of Pharmacoepidemiology and Pharmacoeconomics, Department of Medicine, Brigham and Women’s Hospital and Harvard Medical School, Boston, MA, USA; Program On Regulation, Therapeutics, And Law (PORTAL), Division of Pharmacoepidemiology and Pharmacoeconomics, Department of Medicine, Brigham and Women’s Hospital and Harvard Medical School, Boston, MA, USA

**Keywords:** medication abortion, mifepristone, preemption, public health, US Food and Drug Administration

## Abstract

On June 24, 2022, the Supreme Court issued an opinion in which five justices voted to overturn *Roe v Wade*. Even before the final opinion issued, scholars and advocates had begun to consider legal strategies that might mitigate the decision’s anticipated harmful consequences. One such strategy involves challenging state restrictions on Food and Drug Administration (FDA)-approved pregnancy termination drugs on preemption grounds. This article begins by exploring how these challenges might fare—considering both drug-specific restrictions and complete bans on abortion—arguing that there are compelling legal grounds on which courts should conclude that many state restrictions are preempted. Importantly, although these state restrictions have arisen within a larger debate about reproductive health care, this is far from the only area in which states seek to regulate prescription drugs. States have long regulated drugs in ways that diverge from FDA, arguably increasingly so in recent years. Accordingly, the article investigates the implications that preemption challenges in the abortion context may have for other areas of state drug regulation, making the case that the benefits of public health federalism need not be undermined by successful preemption challenges in the abortion arena.

## I. INTRODUCTION

On June 24, 2022, the Supreme Court issued an opinion in *Dobbs v Jackson Women’s Health Organization* in which five justices voted to overturn *Roe v Wade* and *Planned Parenthood v Casey*, eliminating a right to abortion grounded in the US Constitution.^1^ The decision has had immediate, harmful consequences, which are disproportionately falling on marginalized people.^2^ Emergency rooms in states newly prohibiting abortion care are denying or delaying life-saving care for people experiencing miscarriages or ectopic obstructions.^3^ In July, a 10-year-old rape survivor was denied an abortion in Ohio, requiring her to cross state lines for health care.^4^ Many states are denying millions of people the ability to make decisions to protect their health and well-being,^5^ with greater restrictions and other harms looming on the horizon.^6^

One legal strategy that has gained attention as a promising avenue to mitigate *Dobbs’*s consequences involves challenging state restrictions or bans on Food and Drug Administration (FDA)-approved pregnancy termination drugs as preempted by the Federal Food, Drug, and Cosmetic Act (FDCA).^7^ The same day that the *Dobbs* opinion issued, Attorney General Merrick Garland issued a statement in which he said ‘[T]he FDA has approved the use of the medication Mifepristone. States may not ban Mifepristone based on disagreement with the FDA’s expert judgment about its safety and efficacy’.^8^ Preemption also appears to be one focus of the ‘reproductive rights task force’ that the Department of Justice announced in July 2022.^9^ Even before the *Dobbs* opinion came out (and before a draft of the majority opinion was leaked in May 2022), GenBioPro, a manufacturer of a generic mifepristone product, had challenged Mississippi’s restrictions on the drug,^10^ and scholars—including two authors of this article—had considered preemption challenges to states’ various pre-*Dobbs* restrictions on mifepristone as a possible means to improve access to reproductive health care.^11^

Against this background, this article considers the fate of potential preemption challenges to the new state restrictions on abortion care arising in the wake of *Dobbs*, including both drug-specific restrictions and complete bans on abortion care.^12^ The courts will be the final arbiters. However, there are compelling legal arguments that support courts concluding many state laws limiting or banning access to mifepristone are preempted by FDA regulation.

Critically, any preemption challenges to state medication abortion laws will not happen in a vacuum. Although state mifepristone laws have arisen within the larger debate about reproductive health care, abortion is far from the only area in which states regulate prescription drugs. States have long regulated pharmaceuticals in ways distinct from FDA’s approach,^13^ and federal, state, and local variation may serve important public health goals.^14^ For instance, public health scholars have long viewed state tort law as supplementing FDA oversight by ‘providing an added safeguard in the case of drugs in the market’, thus ‘serving an important public health function’.^15^

There are valid concerns about the impacts of preemption challenges to state medication abortion laws on other areas of state regulation. If medication abortion preemption challenges prove unsuccessful, preemption defenses in tort lawsuits may be weakened, but doors may be opened to state bans on other FDA-approved products—for example, states may be interested in banning COVID-19 vaccines or drugs for HIV pre-exposure prophylaxis (PrEP).^16^ Successful preemption challenges, by contrast, may be used as fodder to strengthen preemption defenses in tort lawsuits, while limiting states’ ability to regulate the use of drugs in areas in which states historically have regulated quite extensively, as with the use of opioid analgesics. Ultimately, we argue that, although these potential for broader effects of preemption challenges to state laws governing abortion drugs are important to consider, preemption challenges need not be understood as risking beneficial public health federalism because FDA and state regulation of mifepristone may be distinguishable from other areas of intersecting federal and state pharmaceutical regulation.

To develop these arguments, the article first discusses current medical knowledge regarding mifepristone and explains how preserving access to mifepristone is one important step that can help mitigate *Dobbs*’s consequences. Next, the article explains that while FDA tightly regulates all prescription drugs, mifepristone has been subject to more federal scrutiny than most. The article then demonstrates why courts could—and should—understand federal law as preempting much state regulation of mifepristone, considering as examples state restrictions on access to mifepristone that fall short of a ban, a medication ban, and a total abortion ban of the kind that has followed the *Dobbs* decision in some states. Finally, the article considers the implications that preemption challenges to state mifepristone restrictions may have for the future of public health federalism beyond the context of abortion.

## II. THE PROMISE OF MIFEPRISTONE ACCESS

In an Executive Order issued on July 8, 2022, President Biden identified access to medication abortion as one of five priorities in his administration’s plan to protect access to reproductive health care after *Dobbs*.^17^ Preserving access to medication abortion, especially where access to procedural abortion care is limited, holds promise and is rightfully a priority because mifepristone^18^—a drug approved and used for medication abortion—is safe, effective, and relatively easy for health care professionals and patients to use.^19^

Mifepristone is a progestin antagonist that FDA initially approved for medication abortion over 20 years ago.^20^ It is used in combination with another drug, misoprostol, to induce abortion.^21^ This regimen is highly efficacious.^22^ In the two trials supporting approval of the drug, 97 per cent (*n* = 16,794) and 96 per cent (*n* = 18,425) of participants taking mifepristone and misoprostol had a complete medication abortion (with efficacy decreasing slightly over the course early pregnancy, from 98 per cent at 7 weeks to 93 per cent in the 10th week).^23^ Mifepristone’s effectiveness—how well a drug works in the real world—is also well documented. One prospective observational study comparing patients who took 200 mg of mifepristone followed by 800 micrograms of misoprostol 24–48 hours later at 57–63 days versus 64–70 days of gestational age found that the effectiveness was 94 and 93 per cent, respectively.^24^ Another study that analyzed 13,373 case reports between 2006 and 2011 for women who took the same regimen up to 63 days gestational age found an overall success rate of 98 per cent.^25^

Mifepristone has also proven extremely safe. Although some women who take the drug experience minor side effects such as nausea, headache, and dizziness,^26^ serious adverse reactions are rare. FDA reported that 0.03–0.5 per cent of women taking mifepristone, in the US clinical studies, required a transfusion, 0.2 per cent had sepsis, and 0.04–0.6 per cent had a related hospitalization. The frequency of these events was similar in non-US clinical studies: 0–0.1 per cent of women required a transfusion, <0.01 per cent had sepsis, and 0–0.7 per cent had a related hospitalization.^27^ In mifepristone’s first 16 years on the US market (2000–16), only 19 deaths were reported to FDA out of more than 3 million women who had taken the drug, making the drug’s associated mortality rate 0.00063 per cent or 14 times lower than the background risk of pregnancy-related death.^28^ Although FDA has long restricted the prescribing and dispensing of mifepristone, mifepristone use in countries without such restrictions has proven equally safe.^29^

Beyond mifepristone’s safety and effectiveness, its use may be harder for states to police than procedural abortion and thus even more important for retaining access to necessary health care in the post-*Dobbs* era. As Professor Greer Donley explained, ‘Historically, abortion was done by procedures, which meant that, if you could control doctors, you could really control abortion provision. But now that abortion pills exist, a state’s abortion ban is not going to have the same effect as it used to’.^30^ Even before *Dobbs*, mifepristone was key to reproductive health care. Many people preferred the privacy of medication abortion, and unlike with procedural abortion, non-physician clinicians can sometimes provide medication abortion.^31^ Already, medication abortion makes up over half of all abortions in the USA, and this proportion will almost certainly increase post-*Dobbs*.^32^

## III. EXTENSIVE FDA REGULATION

Since approving mifepristone in 2000, FDA has subjected mifepristone to more scrutiny and regulation than most other prescription drugs.^33^ A safety program, established as a condition of mifepristone’s approval under Subpart H of 21 CFR Part 314,^34^ restricted dispensing to physicians who attested to the ability to assess the duration of pregnancy, diagnose ectopic pregnancies, and provide surgical intervention or access to medical facilities able to provide blood transfusions in cases of incomplete abortion or severe bleeding.^35^ The program further required patients to receive a medication guide and patients and physicians to sign agreement forms. These measures were intended to ensure awareness of possible rare adverse events from taking mifepristone and safe use conditions.^36^ Finally, the program required in-person ingestion of the drug in the presence of the physician.

The federal approach to regulating mifepristone has evolved over the years. Mifepristone is now subject to a REMS with elements to assure safe use (ETASU). FDAAA amended the FDCA to give FDA the statutory power to require that manufacturers institute REMS for prescription drugs if necessary to ensure that the benefits of a drug outweigh its risks.^37^ REMS can have various components, ranging from a medication guide and communication plan explaining risks and safe use practices to patients and physicians, respectively, to more complex ETASU, such as laboratory testing requirements, patient enrollment in a registry, or restricted distribution of the drug through specialty pharmacies.^38^

In 2008, FDA issued a notice listing drugs, including mifepristone, that were deemed to have in effect an approved REMS, under section 909(b) of FDAAA, as a result of existing safe use conditions.^39^ Danco Laboratories then submitted a proposed REMS for mifepristone, which FDA approved in 2011.^40^ The initial REMS incorporated the original safe use conditions and also required three patient visits to receive mifepristone: the first to receive mifepristone from the physician, the second (on day 3) for the physician to determine whether the termination was complete and to provide misoprostol if it was not, and the third (on day 14) to confirm whether complete termination of the pregnancy occurred.^41^

In 2016, FDA approved a revised indication for mifepristone, extending its approved use from up to 7 weeks of pregnancy to up to10 weeks of pregnancy and changing the dosing regimen, in part to allow for greater flexibility for the timing of misoprostol.^42^ While reviewing the data provided to support the expanded indication—including clinical trials, observational studies, and systematic reviews—FDA also conducted a review of the REMS and authorized several changes.^43^ First, the Medication Guide was removed as a component of the program.^44^ Second, the requirement that mifepristone be dispensed by a physician who attested to certain qualifications was expanded to include non-physician clinicians (eg nurse practitioners).^45^ Third, clinicians were no longer required to report all serious adverse events associated with the drug, such as hospitalizations and transfusions, but instead only deaths.^46^ Finally, the number of required office visits was reduced to one. During this visit, patients received mifepristone, instructions to take misoprostol in the next 24–48 hours, and advice to follow-up between 7 and 14 days after treatment to confirm the complete termination of pregnancy.^47^

In April 2019, nearly 20 years after mifepristone was first marketed in the USA, FDA approved a generic version of the drug manufactured by GenBioPro.^48^ A single, shared REMS program was approved to cover both Mifeprex, Danco’s brand product, and generic products.^49^ This shared system included no substantial changes to the REMS elements.

While the COVID-19 pandemic prompted FDA to relax in-person dispensing requirements for other drugs subject to REMS, FDA initially continued to enforce the in-person visit requirement for mifepristone.^50^ The American College of Obstetricians and Gynecologists and several other parties filed a lawsuit in May 2020 to contest this policy, arguing that the in-person REMS requirement posed an undue burden to patients, substantially limiting access without providing patients with a significant benefit.^51^ In July 2020, the court preliminarily enjoined enforcement of the in-person requirement.^52^ Although the Supreme Court stayed the injunction,^53^ in April 2021, FDA announced that it would continue to decline to enforce the in-person requirement during the pandemic based on its review of the safety of mifepristone use during the injunction.^54^

In addition to the lawsuit that challenged the in-person requirement, in 2017, the American Civil Liberties Union (ACLU) and ACLU of Hawaii filed suit on behalf of a physician and several medical organizations, arguing that the entire REMS was unnecessary and that it violated substantive due process, equal protection rights, and the Administrative Procedure Act.^55^ As a result of the ACLU lawsuit, in May 2021, FDA announced that it would review the entire REMS (not just the in-person requirement).^56^ FDA subsequently announced, in December 2021, that the mifepristone REMS will no longer require that the drug be dispensed in-person through clinics, medical offices, and hospitals. Once the modifications to the REMS are approved, it will include only a certification requirement for dispensing pharmacies and prescribing clinicians and a requirement that patients receive counseling. President Biden’s Executive Order calling on HHS to take additional steps to protect access to abortion care may be cause for hope that FDA will consider additional reductions in the mifepristone REMS requirements or, eventually, a release of the REMS altogether.^57^

## IV. THE CASE FOR PREEMPTION OF STATE MIFEPRISTONE RESTRICTIONS

Because of mifepristone’s potential to help preserve access to safe and effective reproductive health care post-*Dobbs*, legal strategies to increase access to mifepristone are critical. This Part explores one such strategy that flows from the extensive FDA regulation of the drug: challenging state restrictions on mifepristone as preempted by FDA regulation.^58^ Although states generally may regulate concurrently with the federal government, even in areas that are extensively federally regulated, as with pharmaceuticals,^59^ the Supremacy Clause of the US Constitution provides that federal law ‘shall be the supreme Law of the land…’.^60^ Thus, where federal law and state law conflict, federal law preempts state law—state law is ‘without effect’.^61^

To determine whether a federal law preempts a state law, ‘the purpose of Congress is the ultimate touchstone’.^62^ Courts may rely on several theories to conclude that Congress intended federal law to displace state law. The two theories most relevant to the question of whether FDA regulation of mifepristone preempts state restrictions are impossibility preemption, in which parties cannot comply with both state and federal law, and obstacle preemption, when state law is an obstacle ‘to the accomplishment and execution of the full purposes and objectives of Congress’.^63^

In this Part, we investigate how impossibility and obstacle preemption apply to three examples of state restrictions on mifepristone—restrictions on medication abortion that fall short of a total ban, a ban on abortion drugs (or mifepristone), and a ban on all abortion care, whether pharmaceutical or procedural.^64^ We strive to articulate the strongest versions of these preemption arguments, showing that courts would be on firm legal ground concluding that state efforts to regulate mifepristone more stringently than FDA does or to ban the drug are preempted and that there might be actions that FDA could take to bolster such preemption claims. A preemption challenge to a total ban on abortion care presents a more difficult case but nevertheless might still succeed.

We start, however, with a note of caution. Preemption determinations are ultimately made by courts, and even the strongest legal arguments that can be made in favor of the FDCA preempting state mifepristone regulation are not without counterarguments, including that courts ‘start with the assumption that the historic police powers of the States were not to be superseded by the Federal Act unless that was the clear and manifest purpose of Congress’.^65^ Given the politics surrounding abortion, there may be strong practical reasons to doubt that this Supreme Court—and some federal judges in appellate and district courts—would be inclined to agree with even the most compelling legal arguments in favor of preemption.^66^ Nevertheless, because of the gravity of the public health harms resulting from *Dobbs*, and the merits of the legal arguments supporting preemption challenges, such challenges are worth pursuing.

### IV.A. Mifepristone Restrictions Falling Short of a Ban

Before *Dobbs*, certain states hostile to abortion care, such as Mississippi, implemented regulatory schemes for mifepristone that restricted the use of the drug in ways that go beyond what FDA has required under its REMS authorities.^67^ Such restrictions—although likely less appealing to states than direct bans in the post-*Dobbs* era—may persist at least for some time or may be pursued in states where the legislatures are unable to enact bans.^68^ We use Mississippi’s regulatory scheme as an example to demonstrate how preemption challenges might succeed for such restrictions. We consider obstacle preemption arguments most apt. For prescribing and dispensing restrictions that fall short of an outright ban on the drug’s sale, impossibility preemption may be less applicable because the state and federal laws act on different parties—thus it remains technically possible for the drug’s manufacturer to comply with the federally required REMS while doctors and pharmacists comply with the state’s restrictions,.^69^

In 2013, the Mississippi Women’s Health Defense Act went into effect. This law limited the circumstances in which mifepristone could be prescribed or dispensed.^70^ The law’s requirements included a physical examination by a physician prior to prescribing, a 24-hour waiting period before dispensing,^71^ ingestion of the drug at an ‘abortion facility’ in the prescribing physician’s presence,^72^ and a follow-up appointment 14 days after dispensing.^73^

FDA could have mandated the same set of restrictions had it deemed them necessary to ensure that mifepristone’s benefits outweighed its risks. In fact, it did mandate many of these requirements at one point—including in-person dispensing, in-person ingestion, and in-person follow-up—later removing these requirements after considering the evidence.^74^ FDA also could have required that mifepristone only be dispensed with documentation that the patient underwent a 24-hour waiting period.^75^ Yet FDA did not do so.^76^ In 2021, after years of experience—and extensive review, including in litigation—FDA determined that all in-person requirements, including the measures Mississippi imposed, were still not necessary for safe use of the drug.^77^

This tension between Mississippi’s regulatory scheme and FDA’s determinations under its REMS authority give a court good reason to conclude that Mississippi’s scheme is preempted as an obstacle to the purposes of federal law. When deciding to require a REMS for a prescription drug, FDA is statutorily required to weigh the benefits and risks of the drug.^78^ And when FDA requires a restrictive REMS with ‘elements to assure safe use’ like the mifepristone REMS, Congress has required FDA to consider not only the risks and benefits of the drug but also other factors related to drug access and burdens on the health care system.^79^ For example, FDA must consider how implementation of a REMS affects drug access and minimize ‘delays or interruptions’ in care.^80^ By imposing additional restrictions on mifepristone beyond those in the REMS, Mississippi upsets this balancing of benefits, risks, accessibility, and burdens that Congress charged FDA with undertaking in developing the mifepristone REMS.^81^ Although, generally, an agency failing to impose a requirement, or failing to ‘act’, makes a weaker preemption argument, by requiring a REMS, FDA has acted and determined based on its complex balancing of benefits, risks, accessibility, and burdens that no additional safeguards are necessary to ensure mifepristone’s safety and effectiveness, or are possible without being ‘unduly burdensome’ on patient access.^82^ For example, FDA has explained that its December 2021 decision to remove in-person requirements from the mifepristone REMS was because ‘data support modification of the REMS to reduce burden on patient access and the health care delivery system and to ensure the benefits of the product outweigh the risks’.^83^ In Mississippi, the state’s restrictions did hinder patient access, prompting GenBioPro, the manufacturer of a generic mifepristone product, to file suit challenging the state’s restrictions—a suit it later voluntarily dismissed without prejudice.^84^

Should FDA decide to eliminate the mifepristone REMS altogether, as many reproductive health care experts recommend,^85^ state restrictions on the drug would be similarly or possibly even more vulnerable to obstacle preemption challenges.^86^ FDA may only release a REMS upon a determination that it is ‘no longer necessary to ensure a medication’s benefits outweigh its risks’.^87^ A decision to remove a REMS reflects an active determination by FDA that a REMS is not needed for a drug to be safe and effective. Thus, as with requirements that go beyond those in an existing REMS, state restrictions when FDA has released a REMS undermine the uniform drug regulatory scheme that Congress sought to create through enacting the FDCA^88^ and frustrate FDA’s ability to do the complex balancing of safety, effectiveness, health care system burdens, and patient access that Congress has required of the agency in the REMS provisions of the FDCA.^89^

Although these arguments in favor of obstacle preemption are strong, it is important to note that such claims are generally considered to be weaker than impossibility preemption claims^90^ and that there exist counterarguments. For example, in *Wyeth v Levine*, the Supreme Court explained that implied preemption arguments are ‘particularly weak where Congress has indicated its awareness of the operation of state law in a field of federal interest, and has nonetheless decided to stand by both concepts and to tolerate whatever tension there [is] between them’.^91^ The Mississippi law was enacted after Congress added REMS authorities to the FDCA, but various restrictions on abortion care have been in place for many years, and it is difficult to imagine that Congress was not aware that states were legislating in this area—which might indicate that Congress intended some level of state and federal regulation to coexist. Nevertheless, as we have highlighted, there are compelling arguments that support courts’ finding in favor of obstacle preemption challenges to restrictions like those imposed by Mississippi.

Additionally, although courts may not defer to FDA’s own views on whether its REMS authorities preempt similar state restrictions on a drug’s use,^92^ there may be steps that FDA could take to strengthen obstacle preemption challenges, should it wish to do so. For example, although FDA does post information about REMS on its website, FDA could—as applicable—more clearly, publicly, and formally document its reasoning for requiring certain elements and not others in a REMS, removing REMS elements, and releasing a REMS altogether. Such explanations might bolster arguments that state restrictions that go beyond those required in a REMS, or remain after a REMS is released, thwart the balancing of considerations that Congress charged FDA with undertaking in its REMS authorities.

### IV.B. Bans on Mifepristone

Even before *Dobbs*—in fact from the time mifepristone was first approved in 2000^93^—states have shown interest in banning mifepristone or other drugs used for medication abortion.^94^ This interest has only grown following the ruling.^95^ However, when premised on a state legislature’s determinations about the safety and effectiveness of mifepristone, such bans are precisely the state laws that Attorney General Garland identified as particularly vulnerable to preemption challenges.^96^

Texas’s Senate Bill (S.B.) 4, enacted in 2021, provides an instructive example.^97^ S.B. 4 established various reporting requirements and protocols for drugs ‘known to have abortion-inducing properties’, specifically naming Mifeprex—the brand name for mifepristone.^98^ Among other provisions and relevant for a discussion of bans on mifepristone,^99^ S.B. 4 requires physicians to ensure that patients are not beyond a gestational stage of 7 weeks before they are dispensed abortion inducing drugs. Anyone who ‘intentionally, knowingly, or recklessly’ violates the law is subject to possible criminal penalties.^100^ The state legislature explained that it was passing the law in part because it had determined that ‘the failure rate and risk of complications [of drug-induced abortion] increases with advancing gestational age’.

FDA, however, has made a different determination about the safety and effectiveness of mifepristone. Although initially approving the drug for use through 7 weeks of gestation and limiting dispensing to physicians,^101^ FDA determined, in 2016, that mifepristone could be safely and effectively used through 10 weeks of gestation and dispensed by non-physician clinicians—and approved a revised indication.^102^ S.B. 4 directly contradicts this relatively recent FDA determination.^103^

This contradiction, in turn, gives rise to an impossibility preemption argument. S.B. 4 effectively bans manufacturers from selling their drug for use in patients from 8 through 10 weeks of pregnancy despite FDA having approved the drug for that specific use. Manufacturers technically could comply with both state and federal law by declining to sell their product for use in patients more than 7 weeks pregnant. However, in 2013, the Supreme Court specifically rejected this kind of ‘stop-selling’ rationale as saving state tort law from impossibility preemption in *Mutual Pharmaceutical v Bartlett*. In that case, a plaintiff injured by the defendant’s drug asserted design-defect claims under state law regarding the drug’s labeling and composition. The defendant drug manufacturer, Mutual, argued that it was impossible to comply with both state and federal law because, under the FDCA, Mutual could not have made the plaintiff’s proposed changes to the drug’s labeling or composition without first obtaining FDA approval for those changes. The Court agreed, further explaining,

The Court of Appeals’ solution—that Mutual should simply have pulled [its drug] from the market in order to comply with both state and federal law—is no solution. Rather, adopting the Court of Appeals’ stop-selling rationale would render impossibility pre-emption a dead letter and work a revolution in this Court's pre-emption case law.^104^

Laws like Texas’s S.B. 4 banning certain federally approved sales that are grounded in the state’s safety and effectiveness judgments could likewise be understood as preempted as they might make impossible compliance with certain post-marketing obligations, such as adverse event reporting in the mifepristone REMS, given that reporting could be used to support criminal prosecution under state law.^105^

Such an impossibility argument is even more powerful for bans on *all* federally-approved sales of a drug. Just as was the case for the defendant drug manufacturer in *Mutual Pharmaceutical v Bartlett*, a law that effectively bans manufacturers from selling their drug for its approved use for pregnancy termination, or that effectively bans manufacturers from selling the substance mifepristone, would mean that manufacturers must obtain FDA approval of a new indication or a new composition of their drug before selling in the state.^106^ In other words, it would be impossible for the manufacturer to comply with both state and federal law without pulling its drug from the state’s market—which is precisely the solution that the Supreme Court rejected in *Mutual Pharmaceutical v Bartlett*.^107^

Obstacle preemption challenges are also worth consideration.^108^ As noted above, obstacle preemption is seemingly disfavored by some courts as a general matter.^109^ This disfavor might be amplified with respect to state prescription drug bans that contradict FDA approval decisions, specifically, rather than decisions about REMS requirements. Uncodified language included in the 1962 Amendments to the FDCA—the amendments that created the modern approval standard—specified that state authority was preserved except where it ‘direct[ly] and positive[ly] conflict[s]’ with those amendments.^110^ This, in turn, might lend support to arguments that only impossibility theories should be relevant where the conflict between state and federal law concerns the drug approval standard (rather than, for example, the REMS provisions of the FDCA added in separate 2007 amendments).^111^

However, there are strong obstacle preemption arguments. One way that state mifepristone bans may pose an obstacle to the purposes of the FDCA is by eliminating national uniformity in the drug market. As Professor Lars Noah has argued, ‘to the extent that Congress intended for the FDA to make definitive and nationally uniform judgments about the safety and effectiveness of pharmaceutical products, state efforts to second-guess the agency’s determinations certainly would threaten to frustrate those … purposes’.^112^ Although there is no express provision in the FDCA describing national uniformity in the prescription drug market akin to what exists for non-prescription drugs,^113^ the structure of the FDCA, and the history of its enactment, support the view that Congress intended the law to create a nationally uniform market for prescription drugs. For example, scholars have explained that industry sought federal regulation of food and drugs because, in the early 20th century, ‘inconsistencies in applicable state laws made operating on a national scale increasing difficult’.^114^ Similarly, the House Report accompanying the Pure Food and Drug Act of 1906—the predecessor to the FDCA—explained ‘[o]ne of the hoped-for good results of a national law … is the bringing about of a uniformity of laws and regulations on the part of the States within their own several borders’,^115^ and the goal of national uniformity remained a focus in Congress’s deliberations leading to the 1938 enactment of the FDCA.^116^ Moreover, the modern drug approval structure—wherein drug manufacturers must conduct time-consuming, expensive clinical trials to demonstrate safety and effectiveness sufficient to obtain FDA approval—is premised on the promise of a national market being available to those manufacturers who do prove their drug safe and effective.^117^

Another reason might arise from FDA’s congressionally defined mission, which includes both ‘protect[ing] the public health’ ‘by ensuring that … drugs are safe and effective’ as well as ‘promot[ing] the public health’ by ‘taking appropriate action on the marketing of regulated products in a timely manner’.^118^ In *Zogenix, Inc. v Patrick*, the manufacturer of an FDA-approved, extended-release single-ingredient hydrocodone products challenged the Massachusetts ban on the prescribing and dispensing of the drug, and a federal judge pointed to this mission in her decision to enjoin the ban, reasoning that Massachusetts ‘interposed its own conclusion about [the medication’s] safety and effectiveness’ which in turn ‘undermine[d] the FDA’s ability to make drugs available to promote and protect the public health’.^119^ To be sure, FDA cannot ‘guarantee’ any person access to any given drug—for instance, FDA does not regulate prescription drug pricing (and cannot assure patients will be able to afford a drug), nor can the agency compel a manufacturer to continue to make and sell a product if the manufacturer chooses to cease production for business (or any) reasons. Nevertheless, as the judge in *Zogenix* observed, FDA’s public health mission, as described in section 1003 of the FDCA,^120^ includes aspects of drug availability.^121^

Considering these purposes of the FDCA, obstacle preemption arguments apply in a relatively straightforward way to state laws that completely ban mifepristone, analogous to Massachusetts’s total ban on prescribing and dispensing single-ingredient hydrocodone products. Importantly, this holds true even if the state laws are framed as regulating the medical practices of prescribing and dispensing mifepristone—areas conventionally understood to be generally outside FDA’s direct authority and within states’ authority—rather than as laws regulating the drug itself.^122^ In at least two cases—*Zogenix* and a case involving a successful ‘field preemption’ challenge to Maine’s attempt to revise its practice of pharmacy laws to allow the importation of unapproved drugs—federal judges have concluded that states cannot escape preemption challenges simply by framing their efforts to regulate prescription drugs as part of their oversight of medical practice.^123^

The arguments should likewise apply to any partial bans on mifepristone, such as Texas’s S.B. 4. After Massachusetts’s complete ban on prescribing and dispensing single-ingredient hydrocodone products was enjoined, Massachusetts revised its regulations to require prescribers ‘take certain steps before prescribing Zohydro [the only approved extended-release single-ingredient hydrocodone product at the time]’.^124^ In subsequent litigation, the judge declined to dismiss the obstacle preemption challenge to these new regulations because the drug manufacturer’s complaint alleged that the regulations were affecting the drug’s availability, and ‘if the allegations are proven’, the manufacturer would be ‘entitled to relief’.^125^ S.B. 4, and laws like it, clearly restrict the availability of mifepristone—and following the rationale of the subsequent *Zogenix* decision, may therefore be vulnerable to obstacle preemption arguments. FDA’s relatively recent 2016 decision to extend mifepristone’s approved indication from use through 7 weeks, to use through 10 weeks of pregnancy, only strengthens this argument.^126^ To approve the new indication, the agency reviewed the safety and effectiveness data and determined that the benefits of mifepristone outweigh its risks in women seven to 10 weeks pregnant. Texas’s S.B. 4, according to the text of the law itself, likewise is premised on a determination about the safety of mifepristone but with the Texas legislature reaching a different conclusion than FDA did. Accordingly, a court could reasonably conclude that S.B. 4 contradicts, and upsets, the complex balancing of risks and benefits that Congress has charged FDA with conducting in its approval process, undermining the uniform drug regulatory scheme created by the FDCA, the FDA’s mission to promote public health through helping to ensure the availability of safe and effective drugs, or both.

Thus far, we have considered state mifepristone bans premised on state legislatures’ judgments about the drug’s safety and effectiveness. A key question, especially post-*Dobbs*, is whether a state mifepristone ban based on reasoning other than safety and effectiveness concerns would likewise be vulnerable to an impossibility or an obstacle preemption challenge. For example, in February 2022, ‘The Alabama Chemical Abortion Prohibition Act’ was introduced into Alabama’s House, which would make it ‘unlawful for any person or entity to manufacture, distribute, prescribe, dispense, sell, or transfer the “abortion pill,” otherwise known as RU-486, Mifepristone, Mifegyne, or Mifeprex, or any substantially similar generic or non-generic abortifacient drug’ in the state.^127^ The bill includes several proposed findings of the state legislature, one of which is safety-related,^128^ but many of which are not, including explaining that ‘Alabama is committed to the sanctity of human life, from conception to natural death’ and ‘in this state [] abortion is not health care’.^129^ In a post-*Dobbs* world, states may do away with the pretense of including in laws findings on mifepristone’s safety and effectiveness that differ from FDA’s and instead rely on findings framed as social or moral concerns regarding fetal life. At the same time, FDA is not generally authorized to consider the social or moral questions as part of its consideration of a product’s safety and effectiveness,^130^ an interpretation that the agency itself has put forward.^131^

The oft-invoked presumption against preemption^132^—which Professor Elizabeth McCuskey has described as ‘plac[ing] a thumb on the intent scale’ against finding Congressional intent to displace state law^133^—may be the aspect of preemption doctrine that provides the best evidence that states have at least some latitude to regulate in ways that contradict federal law when states are considering factors outside the purview of the relevant federal regulator. This is, perhaps, why Attorney General Garland specifically asserted that states ‘may not ban Mifepristone *based on disagreement with the FDA’s expert judgment about its safety and efficacy*’.^134^

Yet, logically, an impossibility challenge should not turn on the stated purpose of state law—if parties cannot comply with both state and federal law, they are in a bind regardless of whether state law reflects a direct disagreement with the federal government or some other considerations. Consider state tort law. Although, at a high level, the FDCA and state tort law share some similar product-safety aspects, it is also the case that tort law’s purposes are distinct from the FDCA’s purposes. Unlike FDA regulation, which does not provide a private right of action, tort law serves to offer injured people the opportunity for compensation, for example.^135^ Those differing purposes have not stopped courts from concluding that the FDCA preempts state tort law in certain circumstances in which compliance with both is impossible. And just as permitting a stop-selling argument to prevail in tort lawsuits ‘would render impossibility pre-emption a dead letter’, in the Court’s words, so too would allowing states to escape impossibility preemption merely by claiming to be considering some factor outside the federal government’s purview in banning a drug.^136^

Similarly, a state’s purpose in banning mifepristone should not be understood as relevant to obstacle preemption challenges. Because FDA disclaims authority to consider ‘moral, religious, or ethical issues’ regarding the products it regulates, a court, as a practical matter, might be tempted to conclude a state government can do so without frustrating the purposes of FDA oversight.^137^ Contrary to this intuition, however, courts should understand FDA’s lack of authority to consider ‘moral, religious, or ethical issues’ as strengthening preemption challenges to morally-based mifepristone bans that thwart the FDCA’s creation of a nationally uniform drug market. That is, in enacting the FDCA, Congress envisioned a nationally uniform drug market based on technical determinations about drug safety and effectiveness—excluding moral, religious, and ethical considerations from determining the parameters of that uniform market. Likewise, regardless of the state’s purpose, bans on FDA-approved drugs, by limiting their availability, arguably thwart FDA’s mission to promote public health through helping to ensure the availability of safe and effective drugs.^138^

### IV.C. Complete Bans on Abortion Care

In addition to retaining or enacting drug-specific restrictions or bans on mifepristone, post-*Dobbs *states will also, enact, and in some cases already have enacted, complete or near-complete bans on all abortion care. For example, Missouri law now provides ‘no abortion shall be performed or induced upon a woman, except in cases of medical emergency’,^139^ while Ohio prohibits any person from ‘knowingly and purposefully perform[ing] or induc[ing] an abortion’ after a fetal heartbeat has been detected unless the abortion ‘is designed or intended to prevent the death of the pregnant woman or to prevent a serious risk of the substantial and irreversible impairment of a major bodily function of the pregnant woman’.^140^

Although these laws do not specifically target an FDA-approved prescription drug, as mifepristone bans do, they nevertheless have the same impact as a mifepristone-specific ban, effectively prohibiting drug distribution.^141^ Nevertheless, challenges to state bans on all abortion care on the ground that such laws are preempted by the FDCA, at least at first glance, seem less likely to succeed than challenges brought against drug-specific restrictions or bans. As Professor David Cohen, Professor Greer Donley, and Dean Rachel Rebouche have argued, it is relatively easy to imagine courts applying ‘the concept that Congress does not hide huge, politically-relevant policy decisions in the interstices of a statute’ (the ‘no-elephants-in-mouseholes doctrine’) to preemption arguments.^142^ In other words, by enacting the FDCA, which does not ‘explicitly’ mention abortion, Congress did not ‘implicitly’ give FDA the power to decide the social question of whether abortion is permissible.^143^

More broadly, the less state laws appear to be replicating, and thwarting, the careful balancing of benefits and risks that Congress has tasked FDA with for drug approval and REMS decisions, and the more state laws appear to be doing ‘something else’, the weaker courts may perceive preemption arguments to be as a practical matter—particularly when states are regulating medical practice, an area conventionally understood to be within states’ purview.^144^ Perhaps for this reason, abortion opponents have offered medical aid in dying (MAID) as an instructive example.^145^ Ed Whelan, for example, argued ‘[a]ssume that the FDA approved a drug for use in physician-assisted suicide. Why would anyone imagine that FDA approval overrode state laws barring physician-assisted suicide? Why should it be any different [state abortion laws]?’^146^

Notwithstanding this practical intuition, as a legal matter, it may not be the case that states are free to regulate medical practical in ways that effectively ban FDA-approved drugs.^147^ Complete or near-complete state bans on abortion care effectively prohibit the distribution of mifepristone for its FDA-approved use, requiring the drug manufacturer to seek FDA approval of a new indication for its product or to stop selling its product within the state. If the Court’s reasoning in *Mutual Pharmaceutical v Bartlett* is to be taken seriously—that a manufacturer’s ability to stop selling a product within a state’s borders is no counter to an impossibility preemption theory^148^—then, total bans on abortion care are potentially vulnerable to arguments that they make it impossible for drug manufacturers to comply with state and federal laws.^149^ Likewise, the obstacle preemption questions raised by state mifepristone bans may be raised by state bans on all abortion care. State bans on all abortion care will limit the availability of mifepristone, in turn undermining the uniform drug regulatory scheme that Congress envisioned in creating the FDCA as well as FDA’s mission to promote public health through helping to ensure the availability of safe and effective drugs. That is, courts could conclude that, while states retain significant power to regulate the practice of medicine, they do not have the power to regulate medical practice in ways that make compliance with FDA requirements impossible, or obstruct the purposes of the FDCA.

## V. BEYOND ABORTION

As the above analysis demonstrates, preemption challenges to state mifepristone regulation—including both drug-specific restrictions and complete bans on abortion care—hold promise for mitigating the consequences of *Dobbs*. Their success would have clear public health benefits in the context of abortion. States, however, also regulate the uses of prescription drugs for many other indications and have done so since before FDA’s inception.^150^ This regulation might come in the form of positive law, as when Massachusetts attempted to ban the prescribing and dispensing of FDA-approved, extended-release single-ingredient hydrocodone products or California’s 2004 ‘track and trace’ law that sought to impose stricter requirements on the drug supply chain than FDA did.^151^ This regulation also might come in the form of tort liability in which patients harmed by a prescription drug seek compensation for their injuries from drug manufacturers under state law.

Such overlapping state and federal oversight can have public health benefits. For example, Professors Stephanie David and Sara Rosenbaum explained,

“[S]tates also engage in extensive health and welfare regulation, often in ways that overlap with federal law, frequently supplementing federal safeguards. Thus, for example, federal law establishes standards for preventing unsafe products from entering the market, while state laws may add remedies for people who are injured by unsafe products…”^152^

They go on to explain that tort law, in particular, benefits public health by protecting consumers through producing additional information about drugs against the reality that ‘FDA’s resources to monitor drugs on the market are limited, particularly with regard to post-marketing surveillance of approved drugs’.^153^ In this Part, we investigate the effects that preemption challenges to state mifepristone restrictions—whether they fail or succeed—may have for other areas of intersecting state and FDA drug regulation.^154^

### V.A. What If Challenges Fail?

As our analysis in Part IV acknowledges, preemption challenges to state mifepristone restrictions may ultimately prove unsuccessful for various reasons. Depending on a court’s reasoning, such an outcome may weaken preemption defenses in certain tort lawsuits—for example, if a Supreme Court opinion, notwithstanding *Mutual Pharmaceutical*, endorsed a stop-selling rationale as an effective rebuttal to an impossibility preemption argument—which may be viewed as a public health win.^155^ However, opinions in these cases may also be written narrowly, distinguishing tort law from the abortion context, avoiding this result.

Likely more problematic for public health is if failed preemption challenges to state mifepristone laws pave the way for state laws banning or imposing more stringent restrictions than FDA’s on other prescription drugs. This may be a particular risk for drugs for other stigmatized indications or that are otherwise controversial. Interest in limiting or prohibiting access to—or otherwise shaping use of—such drugs is not merely hypothetical. The same advocates that pushed Texas to adopt S.B. 8, which permits private lawsuits against any person who assists a person getting abortion after 6 weeks of pregnancy, are reportedly now seeking to limit insurance coverage for drugs indicated for HIV PrEP.^156^ A bill was introduced into Ohio’s house in 2019 that would ban insurance coverage for hormonal contraceptives or contraceptive devices.^157^ Florida declined to order COVID-19 vaccines for children under 5 years of age, and declined to recommend their administration in that population, in June 2022.^158^ Concerns have also been raised about the possibility that state lawmakers will attempt to restrict access to selective serotonin reuptake inhibitors (SSRIs) and other psychiatric drugs.^159^ Although the previous examples represent measures pursued by conservative activists and lawmakers, interest in such efforts is not limited to one political party. In fall 2020, amidst concerns that FDA bowed to political pressure from President Trump’s Administration to authorize certain COVID-19 therapeutics on questionable scientific evidence, New York established a process by which the state would independently review any FDA-authorized COVID-19 vaccines.^160^ Somewhat similarly, under Governor Deval Patrick, Massachusetts attempted in 2014 to ban the prescribing and dispensing of extended-release single-ingredient hydrocodone products, as discussed in Part IV.

Although these examples—excluding Massachusetts—did not constitute outright bans, one can easily imagine how the same political forces that drove these measures could pursue more severe restrictions or bans on these and other prescription drugs. A patchwork of state judgments diverging from FDA’s would undermine public health by limiting patient access to safe and effective products and by making the market for prescription drugs no longer uniform.^161^ Yet, the possibility that preemption challenges to state mifepristone laws will fail does not militate against pursuing such challenges for several reasons. As explained in Part II, mifepristone access is critical to preserve needed health care. Additionally, there may be ways to distinguish state mifepristone bans and restrictions from state efforts to regulate other prescription drugs. For example, if preemption challenges to state mifepristone bans are unsuccessful because courts find persuasive the idea that states are free to regulate abortion drugs on social policy grounds that FDA does not consider, state efforts to regulate at least certain other prescription products—like potential bans on COVID-19 vaccines or therapies or SSRIs—might not be as easily characterized as grounded in something other than public health or scientific determinations.^162^ But, perhaps most persuasively, states are clearly pursuing restrictions on other prescription drugs^163^ whether mifepristone laws are challenged or not; it, therefore, makes little sense to hold up important public health efforts to increase access to mifepristone for fear states will do something they are already doing.^164^

### V.B. What If Challenges Succeed?

Having considered the possible implications of courts rejecting preemption challenges to state mifepristone laws, we now consider what the effects of courts endorsing these arguments might be. One possibility is that preemption defenses in failure-to-warn or other tort lawsuits will be strengthened. For instance, in *Wyeth v Levine*, a majority of the Supreme Court rejected a preemption defense in a failure-to-warn case brought against the manufacturer of a brand-name drug product but based on facts that happened before Congress added the REMS provisions to the FDCA. Prominent food and drug law attorneys have argued that ‘a preemption defense premised on the presence of a REMS is perfectly intuitive’ for many of the reasons discussed in Part IV.A.^165^ It may be that successful challenges to state mifepristone restrictions, that at least partly rely on FDA’s REMS authorities, will be used by courts as reason to accept preemption defenses in lawsuits involving drugs subject to REMS.^166^

Such an outcome would be troubling from a public health policy perspective. State tort law is an important supplement to FDA oversight of marketed drugs. It is an avenue for learning vital information about drug safety, serves a role separate from FDA’s by compensating injured patients, and can be a catalyst to change industry and government regulators’ behaviors.^167^

Here too, however, there may, be ways to distinguish preemption analyses in the context of state mifepristone restrictions and state tort law. For example, the Ninth Circuit recently declined to extend the Supreme Court’s holding in *Pliva v Mensing*—in which the Court concluded that a failure-to-warn claim against a manufacturer of a generic drug was preempted on an impossibility theory—to drug distributor defendants in a much more similar scenario than is presented by state mifepristone laws.^168^ Moreover, given *Pliva* and a similar holding in *Mutual Pharmaceutical v Bartlett*, claims against manufacturers of generic drugs, which comprise roughly 90 per cent of all drugs dispensed in the USA,^169^ are already often preempted. Against that background, the possibility that preemption challenges to state mifepristone restrictions will further expand preemption defenses in tort lawsuits might not outweigh the potential benefits for reproductive health care access that such challenges hold.

Beyond tort law, successful preemption arguments regarding state mifepristone laws may be extended to other areas in which states often regulate prescribing and dispensing. An obvious example is state regulation of the prescribing and dispensing of opioid analgesics when that regulation comprises requirements that FDA could also impose under the FDCA.^170^ As with state mifepristone restrictions, states heavily regulate the prescribing and dispensing of opioids even though FDA has long required REMS for various classes of opioid analgesics.^171^ Some requirements imposed by states have clearly been considered by FDA. For example, some states, such as Ohio, require that opioid treatment agreements be used with certain patients.^172^ These agreements, also known as pain contracts, opioid contracts, or pain agreements, go beyond informed consent documents, outlining the steps a patient must take to receive their medications. Although FDA has declined to require such an agreement as part of the opioid analgesic REMS (eg by requiring that such an agreement be documented as a condition of safe use), FDA’s Safe Use Initiative developed a model agreement in 2012, which remains available on FDA’s website, suggesting the agency has given thorough consideration to such agreements.^173^ In a scenario like this, where state requirements go beyond those that FDA has chosen to implement in a REMS, opioid restrictions may seem vulnerable to preemption challenges in much the same way mifepristone restrictions may be.^174^

More generally, concluding that FDA’s REMS authorities, in particular, preempt any and all state restrictions that FDA could impose might give some public health experts pause. Public health scholars have critiqued FDA’s use of its REMS authorities, and empirical studies have cast doubt on how well at least certain REMS are helping to mitigate drugs’ serious risks.^175^ The Department of Health and Human Service Office of Inspector General has also critiqued FDA’s implementation of its REMS authorities, both as a general matter and with respect to opioid analgesics specifically.^176^ Regulation supplemental to FDA’s, accordingly, may be beneficial in some circumstances even when FDA has required a REMS.

At the same time, these possible implications of successful preemption challenges to state mifepristone laws do not seem inevitable, or perhaps not even uniformly negative.^177^ As demonstrated in Part III, mifepristone is not only a drug for which FDA has required a REMS, but even among the subset of drugs for which a REMS is required, it is also a drug for which there is unusually extensive documentation (and litigation regarding) the reasons why FDA has required and released specific elements within the REMS. This particularly extensive documentation and debate within the agency, and between the agency and external reproductive health care advocates, may present a path forward for distinguishing state mifepristone laws from other state laws regarding even drugs subject to REMS.

Even if state opioid restrictions were indistinguishable from state mifepristone restrictions, it might still not be worth delaying the potential reproductive health care benefits of challenging state mifepristone laws on preemption grounds. Reproductive health care experts and organizations widely, if not uniformly, view state restrictions on abortion care as unnecessary and harmful.^178^ By contrast, there is greater disagreement about the public health value of state opioid restrictions. Some of these restrictions, such as requirements for opioid treatment agreements, have been widely criticized as unnecessary, stigmatizing, and ineffective at reducing the risks of opioids.^179^ However, those restrictions are still often supported by some experts or organizations.^180^ Additionally, there is likely to be less agreement overall as to whether the risk that such state restrictions would be displaced under preemption theories would harm, or benefit, public health—and much less agreement about what restrictions on opioid analgesics are necessary and sufficient to ensure the safe use of those drugs.

Finally, when courts conclude state restrictions are preempted, state efforts to regulate prescription drugs nevertheless can influence federal policy or decision-making.^181^ For example, when Massachusetts attempted to ban the FDA-approved, extended-release hydrocodone product, the state’s major concern was that FDA approved the drug without an abuse-resistant formulation, meaning the drug could be ‘crushed and inhaled or injected, making the full dose of hydrocodone available immediately’.^182^ As discussed above, a federal judge concluded, in April 2014, the Massachusetts ban was preempted by the FDCA. Yet, by January 2015, the drug company had developed an abuse-deterrent formulation.^183^ Perhaps the drug manufacturer would have developed, and FDA would have approved, such a formulation anyway, but Massachusetts’s efforts to ban the formulation that lacked abuse-deterrent may have hastened such efforts.^184^ Even when courts ultimately conclude state efforts to regulate prescription drugs are preempted by the FDCA, those state efforts might still be impactful, as a practical matter.

## VI. CONCLUSION

Reproductive rights advocates and scholars are rightly pursuing a comprehensive strategy to address the harmful consequences of *Dobbs* and of the state restrictions on abortion care that have followed and will continue to follow the decision.^185^ However, the legal arguments advanced to protect reproductive health care may also have implications for other areas of public health.^186^ These broader consequences are important to try to understand, particularly at this moment when public health authorities seem generally under attack. In this article, we have shown that one approach, challenging state mifepristone laws as preempted by FDA oversight, need not necessarily lead to an expanded FDA preemption footprint into areas of important complementary state public health regulation, like tort law, nor undermine the future of beneficial public health federalism.

## Footnotes

1
*Dobbs v Jackson*, Women’s Health Organization, _ US _ (June 24, 2022).

2 See, eg Bria Peacock, *A Black Abortion Provider’s Perspective on Post-*Roe *America*, 386 N. Engl. J. Med. e70 (2022) (‘Forced births and reproductive exploitation of Black bodies are historical facts, and history often repeats itself. When it does, marginalized people usually suffer the most’.). In this article, we generally use terms like ‘pregnant people’ because people who are not girls or women, such as transmen and non-binary people, can become pregnant, may need abortion access, and may often face significant barriers in accessing adequate reproductive health care. At the same time, we recognize that ‘reproductive rights are undermined precisely because these are issues that affect women’. British Pregnancy Advisory Service (@BPAS1968), Twitter (Oct. 9, 2021 9:30 AM), https://perma.cc/Q6T8-6LH3. See also Jessica Clarke, *Pregnant People?,* 119 Colum. L. Rev. F. 173, 180 (2019) (‘Policymakers, legal reformers, health care providers, and employers who seek to be more inclusive of transgender, nonbinary, and intersex identities and variations ought to take seriously the idea of formally disentangling binary concepts of “sex” and “gender identity” from pregnancy’.); Ruth Colker, *Uninformed Consent*, 101 B.U. L. Rev. 431, 437 (2021) (‘Although this article often uses the expression “pregnant person,” there is little doubt that society’s prejudices and stereotypes against pregnant people are extended to them because, regardless of their gender, they are considered to be women’.).

3 See, eg Selena Simmons-Duffin, *For Doctors, Abortion Restrictions Create an 'Impossible Choice' When Providing Care*, NPR (Jun. 24, 2022), https://www.npr.org/sections/health-shots/2022/06/24/1107316711/doctors-ethical-bind-abortion (accessed Nov. 16, 2022).

4 See, eg Edward Helmore, *10-year-old Rape Victim Forced to Travel from Ohio to Indiana for Abortion*, The Guardian (July 3, 2022), https://www.theguardian.com/us-news/2022/jul/03/ohio-indiana-abortion-rape-victim. See also Marty Schladen, *Affidavits: 2 More Pregnant Minors Who Were Raped Were Denied Ohio Abortions*, Ohio Capital Journal (Sept. 22, 2022), https://ohiocapitaljournal.com/2022/09/22/affidavits-more-pregnant-minors-who-were-raped-denied-ohio-abortions/ (accessed Nov. 16, 2022).

5 See, eg Am. Col. Obstetrics & Gynecology, More Than 75 Health Care Organizations Release Joint Statement in Opposition to Legislative Interference (July 7, 2022), https://www.acog.org/news/news-releases/2022/07/more-than-75-health-care-organizations-release-joint-statement-in-opposition-to-legislative-interference?utm_medium=social&utm_source=twitter&utm_campaign=acog2022-advocacy&utm_content=joint-statement-legislative-interference (accessed November 16, 2022) (hereinafter, ‘2022 ACOG Letter’); Meghan Holohan, *Methotrexate, Used on Autoimmune Diseases, Can Induce Abortion. Some Patients Cannot Get It*, Today (July 8, 2022), https://www.today.com/health/health/methotrexate-abortion-ban-rcna36764 (accessed November 16, 2022) (describing health care systems declining to prescribe and dispense medications for lupus to women who could become pregnant).

6 See, eg David S. Cohen, Greer Donley & Rachel Rebouche, *The Harshest Abortion Restrictions Are Yet to Come*, The Atlantic (July 11, 2022) (anticipating state efforts to ‘stretch the reach of their abortion penalties outside their borders’, to extend civil and criminal punishments to ‘people seeking abortions and the people assisting them, not just [health care] providers’, and to enact ‘legislation [that] will seek to confer the rights of personhood at conception’); Henry T. Greely, *The Death of Roe and the Future of Ex Vivo Embryos*, 9 J.L. Biosciences 1 (2022) (analyzing ‘the effects on the clinical practice of ART and on scientific research if the death of Roe inspires new legislation to protect embryos outside the uterus’); Marc Spindelman, *What ‘Dobbs’ Means for Women’s Equality*, The American Prospect (June 20, 2022), https://prospect.org/justice/what-dobbs-means-for-womens-equality/ (accessed November 16, 2022) (arguing that *Dobbs* signals ‘future decisions that halt, roll back, or even unravel in their entirety the Supreme Court’s constitutional sex equality jurisprudence’).

7 See, eg James M. Beck, Phillip W. Danziger, Andrew R. Hayes & Sarah B. Johanesen, *Federal Preemption and the Post-Dobbs Reproductive Frontier*, Food & Drug L.J. (forthcoming 2023); David S. Cohen, Greer Donley & Rachel Rebouche, *The New Abortion Battleground*, Colum. L. Rev. (forthcoming 2023) (hereinafter, ‘Cohen, Donley & Rebouche’); I. Glenn Cohen, Melissa Murray & Lawrence O. Gostin, *The End of Roe v Wade and New Legal Frontiers on the Constitutional Right to Abortion*, J. Am. Med. Ass’n (2022).

8 Dep’t of Justice, Attorney General Merrick B. Garland Statement on Supreme Court Ruling in *Dobbs v. Jackson Women’s Health Organization* (June 24, 2022), https://www.justice.gov/opa/pr/attorney-general-merrick-b-garland-statement-supreme-court-ruling-dobbs-v-jackson-women-s (accessed November 16, 2022).

9 David Nakamura & Rachel Roubein, *Justice Dept. Announces Task Force to Fight Overreach on Abortion Bans*, Wash. Post (Jul. 12, 2022).

10
*GenBioPro v. Dobbs*, No. 3:20-cv-652-HTW-LRA (S.D. Miss.). In the days after the *Dobbs* opinion issued, a lawyer representing GenBioPro also reportedly indicated that the company may have other plans to challenge state laws because ‘an FDA drug approval should trump any state moves to limit access’. Lauren Gardner, *Abortion Pill Maker Plans Multistate Legal Action to Preserve Drug Access*, Politico (June 29, 2022), https://www.politico.com/news/2022/06/29/abortion-pill-maker-access-legal-00043202?utm_source=STAT+Newsletters&utm_campaign=984c3ecefd-dc_diagnosis_rachel_COPY_01&utm_medium=email&utm_term=0_8cab1d7961-984c3ecefd-150575837 (accessed November 16, 2022).

11 See Cohen, Donley & Rebouche, *supra* note 7; Patricia J. Zettler & Ameet Sarpatwari, *State Restrictions on Mifepristone Access — The Case for Federal Preemption*, 386 N. Engl. J. Med. 705 (2022).

12 We emphasize that we focus on arguments that the FDCA preempts such state laws. There are federal laws other than FDA’s enabling statute that may preempt state abortion laws or aspects of them. For instance, of the Department of Health and Human Services issued a memorandum in July 2022 taking the position that the Emergency Medical Treatment and Active Labor Act (EMTALA) ‘preempts state abortion bans when needed for emergency care’. Dep’t Health & Hum. Servs., Following President Biden’s Executive Order to Protect Access to Reproductive Health Care, HHS Announces Guidance to Clarify that Emergency Medical Care Includes Abortion Services (July 11, 2022), https://www.hhs.gov/about/news/2022/07/11/following-president-bidens-executive-order-protect-access-reproductive-health-care-hhs-announces-guidance-clarify-that-emergency-medical-care-includes-abortion-services.html (accessed November 16, 2022). Texas filed a lawsuit to challenge this position, while the federal government filed a suit against Idaho asserting its abortion restrictions conflict with EMTALA.

13 See, generally, Patricia J. Zettler, *Pharmaceutical Federalism*, 92 Ind. L.J. 845 (2017). For additional discussions of intersecting state and FDA regulation outside the abortion context, see, for example, Lewis A. Grossman, Choose Your Medicine: Freedom of Therapeutic Choice in America 197 (2021); Myrisha S. Lewis, *Innovating Federalism in the Life Sciences*, 92 Temp. L. Rev. 383 (2020); Elizabeth Y. McCuskey, *On Drugs: Preemption, Presumption, and Remedy*, 38 J. Legal Med. 365 (2018); Lars Noah, *State Affronts to Federal Primacy in the Licensure of Pharmaceutical Products*, Mich. St. L. Rev. 1 (2016); Catherine M. Sharkey, *States v FDA*, 83 Geo. Wash. L. Rev. 1609 (2015). *Cf.* Isaac D. Buck, *The Drug (Pricing) Wars: States, Preemption, and Unsustainable Prices*, 99 N.C. L. Rev. 167 (2020) (discussing intersection of federal and state oversight of drug pricing); Thomas J. Hwang, Aaron S. Kesselheim & Ameet Sarpatwari, *Value-Based Pricing and State Reform of Prescription Drug Costs*, 318 J. Am. Med. Ass’n 609 (2017) (same); Ameet Sarpatwari, Jerry Avorn & Aaron S. Kesselheim, State Initiatives to Control Medication Costs—Can Transparency Legislation Help? 374 N. Engl. J. Med. 2301 (2016) (same); Rebecca E. Wolitz, *States, Preemption, and Patented Drug Prices*, 52 Seton Hall L. Rev. 385 (2021) (same).

14 See, eg Elizabeth Y. McCuskey, *Body of Preemption: Health Law Traditions and the Presumption Against Preemption*, 89 Temp. L. Rev. 95, 112 (2016) (‘preemption also can undercut health reform efforts and valuable experimentation at the state level’). See also Marie Boyd, *Preemption & Gender & Racial (In)equity: Why State Tort Law Is Needed in the Cosmetic Context*, 102 B.U. L. Rev. 167, 173–74 (2022) (‘eliminating state tort law as a potential means of redress for people injured by cosmetics—people who may disproportionately be women and members of other excluded groups—may make cosmetics less safe and exacerbate existing inequities’); Erin C. Fuse Brown & Elizabeth Y. McCuskey, Federalism, ERISA, and State Single-Payer Health Care, 168 U. Pa. L. Rev. 389, 389–90 (2020) (‘To restore balance to health care federalism and pave the way for state reforms of all kinds, this paper proposes federal legislative and jurisprudential solutions: amendments to ERISA's preemption provisions, the addition of a statutory waiver, and/or a reinterpretation of ERISA preemption consistent with congressional intent and the presumption against preemption’.); Paul A. Diller, *Why Do Cities Innovate in Public Health? Implications of Scale and Structure*, 91 Wash. U.L. Rev. 1219 (2014) (arguing ‘cities’ smaller scale, concentrated political preferences, and streamlined lawmaking processes facilitate public health innovation’). *Cf.* Abbe R. Gluck & Nicole Huberfeld*, What Is Federalism in Healthcare For?,* 70 Stan. L. Rev. 1689, 1690 (2018) (‘Without a clear conception of the U.S. healthcare system's goals, how can we know which structural arrangements serve it best, much less whether they are working?’); Lindsay F. Wiley et al., *Health Reform Reconstruction*, 55 U.C. Davis L. Rev. 657, 704 (2021) (‘A health system that replaced knee-jerk deference to states with an allocation of responsibility among governmental units according to their legal and logistical capacities to improve public health would harness the power of federalism for good’.).

15 Stephanie David & Sara Rosenbaum, *Wyeth v Levine: Implications for Public Health Policy and Practice*, 125 Pub. Health Rep. 494, 495 (2010).

16 See, eg Johnny Diaz & Noah Weiland, *Forty-Nine States Preordered Vaccine Doses for Very Young Children. Florida Did Not*, N.Y. Times (June 16, 2022); Christopher Wiggins, *Conservative Texas Lawyer Targets PrEP Access After Abortion Rights*, Advocate (July 13, 2022), https://www.advocate.com/news/2022/7/12/conservative-texas-lawyer-targets-prep-meds-after-abortion-rights (accessed November 16, 2022). We primarily focus on prescription drugs in this article, though we do include biologics, like vaccines, that are regulated similarly to prescription drugs. We also note that, should the Supreme Court also eliminate constitutional protections for contraception, as Justice Thomas suggested it should do in his concurring opinion in *Dobbs*, certain states are likely to limit access to hormonal contraceptives (or do more to limit access than they already do). Much of the preemption analysis described in this article would be similar for state restrictions on prescription contraceptive drugs. For over-the-counter drugs, like emergency contraceptives, and contraceptive devices, however, the FDA preemption analysis may be distinct because, unlike for prescription drugs, there are express preemption provisions in FDCA for such products. 21 U.S.C. §§ 360 k, 379r.

17 E.O. on Protecting Access to Reproductive Healthcare Services (July 8, 2022).

18 Although medication abortion is a general term that covers any use of a drug to induce abortion, we focus in this Part—and throughout the rest of the article—on mifepristone specifically, because it is approved for that purpose.

19
*Cf,* Abigail R.A. Aiken, Jennifer E. Starling, James G. Scott & Rebecca Gomperts, *Requests for Self-managed Medication Abortion Provided Using Online Telemedicine in 30 US States Before and After the Dobbs v Jackson Women’s Health Organization Decision*, 328 J. Am. Med. Ass’n 1768 (2022) (‘Requests for self-managed abortion through online telemedicine increased following *Dobbs*’.).

20 U.S. Food & Drug Admin., Mifeprex (Mifepristone) Approval Letter (Sept. 28, 2000) (hereinafter, ‘2000 Mifeprex Approval Letter’), https://www.accessdata.fda.gov/drugsatfda_docs/appletter/2000/20687appltr.pdf (accessed November 16, 2022).

21 U.S. Food & Drug Admin. Mifeprex (Mifepristone) Labeling 1 (Apr. 2019) (hereinafter, ‘2019 Mifeprex Labeling’), https://www.accessdata.fda.gov/drugsatfda_docs/label/2019/020687s022lbl.pdf (accessed November 16, 2022). By blocking progesterone, mifepristone leads the lining of the uterus to break down, making pregnancy unviable. Misoprostol, taken within 48 hours of taking mifepristone, subsequently empties the uterus. Planned Parenthood, *The Facts on Mifepristone* (2019), https://www.plannedparenthood.org/uploads/filer_public/42/8a/428ab2ad-3798-4e3d-8a9f-213203f0af65/191011-the-facts-on-mifepristone-d01.pdf (accessed November 16, 2022).

22 Efficacy refers to the working of a drug in an ideal setting, such as a clinical trial, whereas effectiveness refers to the real-world evidence on the effect of the drug on the disease or condition.

23 2019 Mifeprex Labeling, *supra* note 20, at 13.

24 Beverly Winikoff et al., *Extending Outpatient Medical Abortion Services Through 70 Days of Gestational Age*, 120 Obstetrics & Gynecology 1070, 1072 (2012).

25 Mary Gatter, Kelly Cleland & Deborah L. Nucatola, *Efficacy and Safety of Medical Abortion Using Mifepristone and Buccal Mifepristone Through 63 Days*, 91 Contraception 269, 271 (2015).

26 2019 Mifeprex Labeling, *supra* note 20, at 7.

27
*Id.* at 8.

28 Mifeprex REMS Study Group, *Sixteen Years of Overregulation: Time to Unburden Mifeprex*, 376 N. Eng. J. Med. 790, 791 (2017).

29 See, eg Laura Schummers et al., *Abortion Safety and Use with Normally Prescribed Mifepristone in Canada*, 386 N. Engl. J. Med. 57, 57 (2022) (comparing 195,183 abortions performed in Canada before mifepristone was available and 84,032 after its availability without any REMS-like restrictions concluding that ‘the abortion rate remained relatively stable, the proportion of abortions provided by medication increased rapidly, and adverse events and complications remained stable’).

30 Lauren Collins, *The Complicated Life of the Abortion Pill*, New Yorker (July 5, 2022). *But cf.* Kayte Spector-Bagdady & Michelle Mello, *Protecting the Privacy of Reproductive Health Information after the Fall of Roe v Wade*, 3 JAMA Health Forum e222656 (2022) (explaining that, unlike before *Roe*, today ‘information generated from a person’s online activity could be used to show that they sought an abortion or helped someone do so’); Leah R. Fowler & Michael R. Ulrich, *Femtechnodystopia*, Stan. L. Rev. (forthcoming 2023) (discussing the post-*Dobbs* privacy risks of Femtech, described as ‘a category of consumer technology addressing an array of “female” health needs’). For additional scholarly analysis of the policing and control of medication and procedural abortion care, see Greer Donley & Rachel Rebouche, *The Promise of Telehealth for Abortion*, in Diagnosing the Home (I. Glenn Cohen, ed.) (Cambridge University Press, forthcoming 2023).

31 See, eg Sarah McCammon & Jonathan Franklin, *FDA Relaxes Controversial Restrictions on Access to Abortion Pill by Mail*, NPR (Dec. 16, 2021), https://www.npr.org/2021/12/15/1064598531/the-fda-could-permanently-lift-some-restrictions-on-abortion-pills (accessed November 16, 2022); Rebecca H. Stone & Sally Rafie, *Medication Abortion: Advocating for Mifepristone Dispensing by Pharmacists*, 104 Contraception 31, 31 (2021) (noting, in 2016, FDA removed requirements for physician-only prescribing).

^32^
See, eg Aiken et al., *supra* note 19; Cohen, Murray & Gostin, *supra* note 7; Lewis Grossman, *Pushing Back with Pills — Enhancing Access to Reproductive Health Drugs after* Dobbs, 387 N. Engl. J. Med. 1056 (2022)

33 As of Mar. 3, 2022, FDA reported that there were 62 active REMS and 297 approved since the Food and Drug Administration Amendments Act (FDAAA) was enacted in 2007. For other data on approved REMS, see U.S. Food & Drug Admin., FDA Risk Evaluation and Mitigation Strategy (REMS) Public Dashboard, https://fis.fda.gov/sense/app/ca606d81-3f9b-4480-9e47-8a8649da6470/sheet/dfa2f0ce-4940-40ff-8d90-d01c19ca9c4d/state/analysis (accessed November 16, 2022). See also Mifeprex REMS Study Group, *supra* note 27, at 790 (noting that in 2016, of approximately 1750 drug and biologic active ingredients approved by FDA, FDA required REMS for only 74); Greer Donley, *Medication Abortion Exceptionalism*, 107 Cornell L. Rev. 627, 640 (2022) (explaining that as of 2022, ‘there [were] only sixty-one REMS programs, covering less than 5% of all FDA-approved drugs’.).

34 Notably, questions have been raised about the appropriateness of approving mifepristone under this pathway, as Subpart H applies only to drugs indicated for a serious or life-threatening illness that offers a clinically meaningful benefit over existing therapies. See, eg Lars Noah, *A Miscarriage in the Drug Approval Process? Mifepristone Embroils the FDA in Abortion Politics.* 36 Wake Forest L. Rev. 571, 580–81 (2001) (‘Mifepristone’s eligibility to use such procedures remains somewhat of a mystery: the drug did not provide the type of therapeutic benefit over existing treatments for a serious illness that the regulations contemplated as justifying an expedited review process’.). FDA concluded that termination of an unwanted pregnancy should be considered a serious condition and that mifepristone provided a clinically meaningful benefit over alternative therapies by allowing patients to avoid a procedure. See U.S. Gov’t Accountability Office, GAO-08-751, Approval and Oversight of the Drug Mifeprex 6, 22 (2008), https://www.gao.gov/assets/gao-08-751.pdf (accessed November 16, 2022).

35 2000 Mifeprex Approval Letter, *supra* note 19; Julie Dohm & Mingham Ji, *An Introduction to Risk Evaluation and Mitigation Strategies*, 104 Contraception 4, 6 (2021).

36 For the medication guide, see the 2005 label for Mifeprex (mifepristone), available at https://www.accessdata.fda.gov/drugsatfda_docs/label/2005/020687s013lbl.pdf (accessed November 16, 2022). For the patient agreement form, see Danco Laboratories, *Patient Agreement, Mifeprex (mifepristone) Tablets* (2000), available at: http://wayback.archive-it.org/7993/20161024033544/http://www.fda.gov/downloads/Drugs/DrugSafety/PostmarketDrugSafetyInformationforPatientsandProviders/ucm111361.pdf (accessed November 16, 2022). For the prescriber guide, see Danco Laboratories, *MIFEPREX (Mifepristone) Tablets, 200 mg, Prescriber’s Agreement* (2000), available at: http://wayback.archive-it.org/7993/20161024033544/http://www.fda.gov/downloads/Drugs/DrugSafety/PostmarketDrugSafetyInformationforPatientsandProviders/ucm111364.pdf (accessed November 16, 2022).

37 FDAAA, Pub. L. No. 110–85, 121 Stat. 823, 926 (codified at 21 U.S.C. § 355–1(a)(1)(E)).

38 21 U.S.C. § 355–1(e)–(f). See also Beatrice L. Brown, Susan F. Wood, & Ameet Sarpatwari, *Ensuring Safe Access to Mifepristone During the Pandemic and Beyond*, 174 Annals Internal Med. 105 (2021) (discussing REMS authorities generally and the mifepristone REMS specifically).

39 Identification of Drug and Biological Products Deemed to Have REMS for Purposes of FDAAA, 73 Fed. Reg. 16,313 (Mar. 27, 2008).

40 Kaiser Family Foundation, The Availability and Use of Medication Abortion (June 16, 2021), https://www.kff.org/womens-health-policy/fact-sheet/the-availability-and-use-of-medication-abortion/ (accessed November 16, 2022).

41 U.S. Food & Drug Admin., 2011 Mifeprex (Mifepristone) REMS, https://www.accessdata.fda.gov/drugsatfda_docs/rems/Mifeprex_2011-06-08_Full.pdf (accessed November 16, 2022).

42 U.S. Food & Drug Admin., Mifeprex (Mifepristone) Approval Letter 1 (Mar. 29, 2016) (hereinafter, ‘2016 Mifeprex Approval Letter’), https://www.accessdata.fda.gov/drugsatfda_docs/appletter/2016/020687Orig1s020ltr.pdf (accessed November 16, 2022).

43
*Id.* at 2.

44 U.S. Gov’t Accountability Office, GAO-18-292, Information on Mifeprex Labeling Changes and Ongoing Monitoring Efforts 8 (2018), https://www.gao.gov/assets/gao-18-292.pdf (accessed November 16, 2022). The Medication Guide remained part of the approved labeling for the drug. This change was consistent with FDA’s overall policy regarding the inclusion of Medication Guides as REMS elements. See U.S. Food & Drug Admin., Guidance: Medication Guides—Distribution Requirements and Inclusion in REMS 7 (Nov. 2011) (‘While all Medication Guides must meet the standard and requirements in part 208, not every newly required Medication Guide will be an element of a REMS’.).

45
*Id.*

46
*Id.*

47
*Id.* at 7.

48 U.S. Food & Drug Admin., Mifepristone ANDA Approval Letter (Apr. 2019), https://www.accessdata.fda.gov/drugsatfda_docs/appletter/2019/091178Orig1s000ltr.pdf (accessed November 16, 2022).

49 U.S. Food & Drug Admin., Mifeprex (mifepristone) Information (Dec. 16, 2021), https://www.fda.gov/drugs/postmarket-drug-safety-information-patients-and-providers/mifeprex-mifepristone-information (accessed November 16, 2022).

50 See, eg Donley, *supra* note 32, at 650 (‘When the FDA under the Trump administration temporarily suspended the in-person requirements of other medications' REMS, including opioids, but refused to do the same for mifepristone, a medical organization sued the FDA and won a preliminary injunction’.). For a further discussion of FDA’s exceptional treatment of mifepristone throughout the drug’s history and including during the pandemic, see also Eli Y. Adash, Rohit S. Rajan, Daniel P. O’Mahony, and I. Glenn Cohen, *The Next Two Decades of Mifepristone at FDA: History as Destiny* 109 Contraception 1 (2022).

51 Compl., Am. Coll. of *Obstetricians & Gynecologists v. United States Food & Drug Admin.*, No. 8:20-CV-01320-TDC 5 (D. Md. 2020) (‘By making life-threatening viral exposure risks a condition of treatment for medication abortion and miscarriage care, the FDA’s continued maintenance of the Mifepristone In-Person Dispensing Requirement jeopardizes the safety of patients, clinicians, and the public at large, with no countervailing benefit—and with particularly severe implications for low-income people and people of color, who comprise a disproportionate share of impacted patients and who are already suffering and dying from COVID-19 at substantially higher rates’.), https://www.acog.org/-/media/project/acog/acogorg/files/advocacy/acog-v-fda-complaint-mifepristone-covid19.pdf?la=en&hash=2C5C6C65F3E6C8A693ACD649C7C12129 (accessed November 16, 2022).=.

52
*Am. Coll. of Obstetricians & Gynecologists v. United States Food & Drug Admin.*, 472 F. Supp. 3d 183 (D. Md. 2020).

53
*Food & Drug Admin. v. Am. Coll. of Obstetricians & Gynecologists*, 141 S. Ct. 578 (2021). See also *id.* at 579 (Roberts, C.J., concurring opinion) (‘courts owe significant deference to the politically accountable entities with the “background, competence, and expertise to assess public health”’).

54 ACLU, FDA Response to ACOG April 2021 (Apr. 12, 2021), https://www.aclu.org/letter/fda-response-acog-april-2021 (accessed November 16, 2022).

55 Compl., Chelius v. Wright, No. 1:17-CV-00493 59–62 (D. Haw. 2017), https://www.aclu.org/legal-document/chelius-v-azar-complaint (accessed November 16, 2022).

56 ACLU, FDA Announces Long Sought-After Review of Harmful Restrictions on Medication Abortion (May 7, 2021), https://www.aclu.org/press-releases/fda-announces-long-sought-after-review-harmful-restrictions-medication-abortion (accessed November 16, 2022).

57 E.O. on Protecting Access to Reproductive Healthcare Services (July 8, 2022).

58 FDA oversight of mifepristone raises this preemption question. But the FDA preemption question is only one of many issues arising in what Professor David Cohen, Professor Greer Donley, and Dean Rachel Rebouche have termed the ‘interjurisdictional abortion wars’ that are following *Dobbs*. For a full discussion of such issues, see Cohen, Donley & Rebouche, *supra* note 7.

59 See, eg *Gregory v. Ashcroft*, 501 U.S. 452, 457 (1991). See also *Cipollone v. Liggett Grp.*, Inc., 505 U.S. 504, 516 (1992) (a court’s preemption analysis ‘start[s] with the assumption that the historic police powers of the States [are] not to be superseded … unless that [is] the clear and manifest purpose of Congress’); Peter Barton Hutt et al., Food & Drug Law: Cases and Materials 424–54, 1342–71, 1746–60 (5th ed. 2022) (discussing FDA preemption case law).

^60^
U.S. Const. art. VI, cl. 2.

61 See, eg *Cipollone*, 505 U.S. at 516.

62
*Wyeth v. Levine*, 555 U.S. 555, 565 (2009).

^63^

*Hines v. Davidowitz*, 312 U.S. 52, 66 (1941). *Cf.* Cong. Rsch. Serv., R45825 Federal Preemption: A Legal Primer (2019) (hereinafter, ‘CRS Federal Preemption’) (stating the Court has warned against using obstacle preemption to strike down state laws that create tension with federal schemes). We do not consider express preemption and field preemption in detail because the FDCA lacks an express preemption provision for prescription drugs and, outside the context of foreign commerce, courts have generally been disinclined to conclude that Congress intended FDA to wholly occupy the field of drug regulation, particularly given state law’s historical role in products liability. See, eg, Cohen, Donley & Rebouche, *supra* note 7; Hutt et al., *supra* note 63, at 424–54; Zettler, *Pharmaceutical Federalism*, *supra* note 13, at 867–70.

64 This article does not examine drug-specific state bans on misoprostol. Although misoprostol is not approved for abortion, as discussed in Part II, the drug is used for medication abortion. Nevertheless, state misoprostol bans may be less politically feasible because the drug has well-known non-abortion uses (eg it is approved for reducing the risk of NSAID-induced gastric ulcers).

65
*Wyeth*, 555 U.S. at 564 (internal quotations and citations omitted). See also Cohen, Donley & Rebouche, *supra* note 7 at 54–58 (discussing some of the weaknesses of FDCA preemption challenges to state mifepristone restrictions, including the political questions doctrine); Zettler, *Pharmaceutical Federalism*, *supra* note 13, at 867–70 (discussing some of the weaknesses of preemption challenges to state regulation of prescription drugs).

66 See, eg Hank Greely (@HankGreelyLSJU), Twitter (June 24, 2022, 3:20 PM), https://twitter.com/HankGreelyLSJU/status/1540414561009905664 (accessed November 16, 2022) (‘my own cynical/realist view is that [FDA preemption] arguments won’t matter to a majority of this Supreme Court’). See also Eric Segall, Supreme Myths: Why the Supreme Court Is Not a Court and Justices Are Not Judges (2012) (arguing that the Supreme Court is a political body making policy).

67 In addition to being approved for pregnancy termination, mifepristone is also approved at a different dosage for Cushing’s Syndrome and marketed under the brand-name Korlym for that use. U.S. Food & Drug Admin., Korlym Labeling, https://www.accessdata.fda.gov/drugsatfda_docs/label/2019/202107s008lbl.pdf (accessed November 16, 2022). Although, as far as we are aware no state has done so yet, it is possible that some states will seek to prohibit off-label prescribing and dispensing of Korlym specifically. Before FDA expanded the pregnancy termination indication for mifepristone from 7 to 10 weeks, several states required on-label use of the drug even though physicians commonly prescribed through 10 weeks of gestation. See, eg Zettler, *Pharmaceutical Federalism*, *supra* note 13, at 900. State restrictions on off-label, rather than on-label, prescribing and dispensing generally pose somewhat different questions than those that are the focus of this article, and often present weaker cases for preemption because off-label uses, are typically uses that FDA has not evaluated and approved. Restrictions on prescribing and dispensing Korlym for pregnancy termination, however, would present what might be an unusually compelling case for preemption. Korlym, as noted above, contains mifepristone as its active ingredient and therefore it seems reasonable to anticipate that it could be used off-label for pregnancy termination. FDA has not required a REMS for Korlym, while a REMS is required for mifepristone approved for pregnancy termination, suggesting FDA has determined that restrictions that would limit Korlym’s off-label use for pregnancy termination are not needed for the benefits of the drug to outweigh its risks. Moreover, FDA has considered the safety and effectiveness evidence for the relevant off-label use, albeit for a different dosage than Korlym contains. Nevertheless, possible restrictions on off-label prescribing and dispensing of Korlym seems somewhat different than the other examples we are considering, and we do not address preemption challenges to such restrictions in detail.

68 For example, the Pennsylvania legislature is reportedly interested in an abortion ban, but one has not passed because the current governor would veto it. See, eg Abigail Tracy, *‘Democrats Have to Get Out of Their Own Way’: Pennsylvania’s Next Governor Will Decide on Abortion Rights*, Vanity Fair (July 12, 2022). Pennsylvania, however, has other restrictions on abortion. See, eg Guttmacher Institute, State Facts about Abortion: Pennsylvania, https://www.guttmacher.org/fact-sheet/state-facts-about-abortion-pennsylvania (accessed November 16, 2022).

69 As one of us has explained elsewhere, ‘[a]lthough medical practitioners ultimately carry out many … REMS requirements, the requirements apply only to drug manufacturers. Thus, regardless of their content, [state] restrictions on the use of [drugs]—which apply to medical practitioners—do not make it “impossible” for any party to comply with both state and federal requirements’. Zettler, *Pharmaceutical Federalism*, *supra* note 13, at 874. *But cf.* Lars Noah, *State Regulatory Responses to the Prescription Opioid Crisis: Too Much to Bear?*, 124 Dick. L. Rev. 633, 667 n.36 (2020) (‘now that the FDA has imposed REMS requirements on most opioids, conflict preemption might operate more strongly than it does for other brand-name drugs’).

^70^
Miss. Code Ann. § 41–41-107.

^71^
Miss. Code Ann. § 41–41-33.

^72^
Miss. Code Ann. § 41–75-1(f). Because of Mississippi’s concentrated attack on abortion rights, only one abortion facility remained in the state before *Dobbs*, and it has now closed. See, eg *Mississippi’s Only Abortion Clinic Has Closed its Doors for Good*, NPR (July 7, 2022), https://www.npr.org/2022/07/07/1110222245/mississippis-only-abortion-clinic-has-closed-its-doors-for-good (accessed November 16, 2022).

^73^
Miss. Code Ann. § 41-41-107.

74 See, eg Cohen, Donley & Rebouche, *supra* note 7; Donley, *supra* note 32; Brown, Wood & Sarpatwari, *supra* note 37.

75 21 U.S.C.§ 355–1(f)(3)(D).

76 For a discussion of the requirements in the REMS, see Part III, *supra*.

77 See Part III, *supra*. In the litigation brought by GenBioPro, Mississippi argued that, regardless of FDA determinations, 18 U.S.C. §§ 1461 and 1462—so-called Comstock laws—federally prohibit the mailing of drugs intended for abortion. Therefore, according to Mississippi, prohibitions on mailing mifepristone cannot conflict with federal law. Memo in Opp. to Mot. For Leave to File Amended Compl., 15–16, No, 3:20-CV-652-HTW-LGI (S.D. Miss.). However, as David Cohen has explained, ‘[T]he circuit courts long ago declared that [14 U.S.C. §§ 1461 & 1462] only apply to unlawful items. So, since abortion is legal and the FDA has approved distribution of mifepristone as safe and effective, including by mail, these are lawful activities and items, thus the law, as interpreted by the courts, does not apply. Even now that some states have made abortion illegal, the pills still are legal under federal law. This is why no one in any of the litigation, regulatory comments, or other advocacy around medication abortion for the past decades has raised these laws, even the Trump administration in the litigation about mailing pills that went to the Supreme Court’. Jonathan H. Adler, *The Next Abortion Battlegrounds*, The Volokh Conspiracy (June 27, 2022), https://reason.com/volokh/2022/06/27/the-next-abortion-battlegrounds/ (accessed November 16, 2022).

^78^
21 U.S.C. § 355–1(a)(1).

79
*Id.* at §355–1(f)(2).

^80^

*Id.* at §355–1(f)(2)(D).

^81^
Zettler & Sarpatwari, *supra* note 11, at 2.

^82^
21 U.S.C. §355–1(f)(2)(D). See also Cohen, Donley & Rebouche, *supra* note 7; Zettler, *Pharmaceutical Federalism*, *supra* note 13, at 875.

83 U.S. Food & Drug Admin., Questions and Answers on Mifeprex, https://www.fda.gov/drugs/postmarket-drug-safety-information-patients-and-providers/questions-and-answers-mifeprex (accessed November 16, 2022).

^84^

*GenBioPro v. Dobbs*, No. 3:20-CV-652-HTW-LRA (S.D. Miss.). There may be other framings of obstacle preemption to state restrictions as well. For example, state requirements that parallel a REMS may thwart FDA’s ability to undertake its statutory obligation to periodically review REMS with ETASU to ensure such requirements ‘assure safe use ... [and] are not unduly burdensome on patient access’. 21 U.S.C. § 355–1(f)(5)(B). If states retain parallel requirements that FDA then determines are no longer necessary, FDA arguably cannot meaningfully revise REMS requirements as envisioned in the statute.

^85^
See, eg Am. Col. Obstetrics & Gynecology, *Improving Access to Mifepristone for Reproductive Health Indications* (June 2018), https://www.acog.org/clinical-information/policy-and-position-statements/position-statements/2018/improving-access-to-mifepristone-for-reproductive-health-indications; (accessed November 16, 2022) Brown, Wood & Sarpatwari, *supra* note 37; Donley, *supra* note 49.

^86^
Zettler & Sarpatwari, *supra* note 11, at 2.

^87^

*Frequently Asked Questions (FAQs) about REMS*, FDA.gov (Jan. 26, 2018), https://www.fda.gov/drugs/risk-evaluation-and-mitigation-strategies-rems/frequently-asked-questions-faqs-about-rems (accessed November 16, 2022). FDA may modify a REMS for similar reasons. 21 U.S.C. § 355–1(g)(4)(B).

88 See, eg Noah, *supra* note 33. See also Hutt et. al, *supra* note 62, at 429 (‘Early support for comprehensive federal food and drug legislation was driven largely by the desire for uniformity. In an 1879 address to the Medical Society of the State of New York, Dr E. R. Squibb advocated the enactment of a nationwide food and drug law, asserting: “It is self-evident that a law to be most effective in preventing the adulteration of food and medicine should be general or national in order to secure universality and uniformity of action....”’).

89 Zettler, *Pharmaceutical Federalism*, *supra* note 13, at 874–76 (making a similar argument regarding state restrictions on certain opioids). See also Jennifer L. Bragg & Maya P. Florence, *Life with A REMS: Challenges and Opportunities*, 13 J. Health Care L. & Pol’y 269, 275–78 (2010) (considering REMS-based preemption arguments); Cohen, Donley & Rebouche, *supra* note 11 (same); Hutt et al., *supra* note 62, at 448 n.4 (same). To the extent FDA is required to consider whether a REMS is needed any time it approves a prescription drug, a similar argument might be made for any approved drug that lacks a REMS. See 21 U.S.C. §355–1(a). That said, a failure to require a REMS at all may be more akin to the kind of federal inaction where preemption is disfavored, particularly as there is likely to be less evidence that FDA carefully considered the variety of factors it must consider for REMS with ETASU.

90 See, eg CRS Federal Preemption, *supra* note 62. But, see Nathan A. Brown & Eli Tomar, *Could State Regulations Be the Next Frontier for Preemption Jurisprudence? Drug Compounding as a Case Study*, 71 Food & Drug L.J. 271, 285 (2016) (arguing that in the FDA space courts are increasingly ‘willing to strike state regulations that are not impossible to abide, but which complicate industry's compliance with an overarching federal program’).

91
*Wyeth v. Levine*, 555 U.S. 555, 575 (2009).

92 See, eg *Wyeth*, 555 U.S. at 576–77 (2009) (declining to defer to FDA’s view on whether its drug labeling requirements preempt state failure-to-warn claims); Catherine M. Sharkey, *The Administrative State and the Common Law: Regulatory Substitutes or Complements?*, 65 Emory L.J. 1705, 1730 (2016) (discussing judicial views on deference to agency positions on preemption); Catherine M. Sharkey, *Preemption by Preamble: Federal Agencies and the Federalization of Tort Law*, 56 DePaul L. Rev. 227 (2007) (same). But see *Wyeth*, 555 U.S. at 625 (Alito, J., dissenting) (criticizing the majority for not ‘relying on the FDA's explanation of its own regulatory purposes’ in its preemption analysis).

93 Shortly after FDA initially approved the drug, a bill was introduced in Oklahoma that would have banned the prescribing, dispensing, and distribution of the drug. H.B. 1038, 48th Leg., 1st Sess (Okla, 2001).

94 See, eg Zettler & Sarpatwari, *supra* note 11.

95 Cohen, Donley & Rebouche, *supra* note 11.

96 Garland Statement, *supra* note 8. But see Noah, *supra* note 13, at 3 (arguing state bans on FDA-approved drugs are not always impermissible).

97 Although S.B. 4 is not a complete ban on mifepristone, we offer it as an example of a ban—rather than an example of mifepristone restrictions discussed in the previous section—because it constitutes a ban on FDA-approved uses of the drug rather than REMS-like restrictions. That is, it provides an example of a state law that is premised on the state’s disagreement with FDA’s approval decision, rather than FDA’s REMS decision, and an example of a state doing so on the ground that, contrary to FDA’s approval decision, the drug is not safe and effective for its approved uses.

^98^
The provisions of interest were codified in Tex. Provision of Abortion-Inducing Drug Code Ann. § 171.063.

99 For example, the law also prohibits manufacturers, suppliers, physicians, or anyone else from mailing mifepristone to a patient. To the extent this differs from what FDA permits under the REMS, this provision may be vulnerable to preemption challenges, consistent with the analysis regarding Mississippi’s regulatory regime *supra*.

^100^

Tex. Provision of Abortion-Inducing Drug Code Ann. § 171.063.

101 2000 Mifeprex Approval Letter, *supra* note 19.

^102^
See 2016 Mifeprex Approval Letter, *supra* note 41.

^103^
See Part II, *supra*, for a discussion of the evidence demonstrating mifepristone’s safety and effectiveness.

104
*Mut. Pharm. Co. v. Bartlett*, 570 U.S. 472, 475 (2013). See also *PLIVA, Inc. v. Mensing*, 564 U.S. 604 (2011) (finding impossibility preemption notwithstanding plaintiffs’ stop-selling arguments); Mayor and City Council of Baltimore v. GlaxoSmithKline, LLC, No. 24-C-20-004788, 2022 WL 537004, at ^*^4 (Md.Cir.Ct. Jan. 28, 2022) (finding preempted the City of Baltimore’s claims when ‘[a]ssuming the truth of the City's allegations, the Generic Manufacturer Defendants would violate their duty under Maryland law by simply marketing ranitidine in its FDA-approved form and with its FDA-approved label. In other words, they would have been required to stop selling ranitidine’.).

105 But see *Ulleseit v. Bayer Healthcare Pharms. Inc.*, No. 19–15778, 2021 WL 6139816, at ^*^1 (9th Cir. Dec. 29, 2021) (declining to extend the preemption analysis in *Mutual Pharmaceutical* to failure-to-warn claims brought against drug distributors). Although following *Dobbs* a law in Texas has gone into effect that criminalizes performing any abortion unless the pregnant patient is facing a life-threatening physical condition, S.B. 4, and other bans on mifepristone, are still useful to consider. For example, depending on the wording of a mifepristone ban, it may prohibit off-label uses of the drug. As another example, states may retain mifepristone bans, even after implementing bans on providing abortion care more generally because drug-specific bans may make criminalizing certain kinds of conduct, such as self-managed abortions, easier.

106 These are intended as examples of the ways that manufacturers may find it impossible to comply both with state bans and FDCA requirements and not necessarily an exhaustive list. For example, similar to S.B. 4, some post-marketing reporting requirements—like the requirement in the mifepristone REMS that deaths associated with use of the drug be reported—might at least arguably be impossible to comply with in states with bans, where reporting might also bring the risks of criminal prosecution. As another example, a partial ban, like S.B. 4, shrinks a manufacturer’s ability to sell its drug for the federally approved indication, and, thus, a manufacturer would be required to seek FDA approval for a new indication from FDA to retain its original market share, potentially creating an impossibility.

107
*Mut. Pharm. Co*, 570 U.S. at 475.

108 See, eg Cohen, Donley & Rebouche, *supra* note 7 (discussing same).

^109^
eg CRS Federal Preemption, *supra* note 62.

^110^
Drug Amendments of 1962, Pub. L. No. 87–781, sec. 202, 76 Stat. 780, 793.

111 See *Wyeth v. Levine*, 555 U.S. 555, 567 (2009) (‘The 1962 amendments added a saving clause, indicating that a provision of state law would only be invalidated upon a “direct and positive conflict” with the FDCA ... Consistent with that provision, state common-law suits “continued unabated despite ... FDA regulation”’.) (internal citations omitted). See also Noah, supra note 13, at 8–9 (discussing the same); Zettler, *Pharmaceutical Federalism*, *supra* note 13, at 869 (discussing the same).

112 Noah, *supra* note 13, at 12.

113 21 U.S.C. § 379r (‘National uniformity for nonprescription drugs’).

114 Ilyse D. Barkan, *Industry Invites Regulation: The Passage of the Pure Food and Drug Act of 1906*, 75 Am. J. Pub. Health 18, 20 (1985). See also David F. Cavers, *The Food, Drug, and Cosmetic Act of 1938: Its Legislative History and Substantive Provisions*, 6 L. & Contemp. Problems 2, 32 (1939) (describing the ‘purpose’ of certain of the FDCA’s drug provisions as ‘assuring uniformity’); Hutt et al., *supra* note 62, at 7–8 (‘national uniformity was an important argument in favor of federal legislation’); Peter Barton Hutt, *The Evolution of Federal Regulation of Human Drugs in the United States: An Historical Essay*, 44 Am. J.L. & Med. 403 (2018) (generally describing the development of FDA drug regulation); Zettler, *Pharmaceutical Federalism*, supra 13, at 852 (‘Federal regulation emerged, in part, as a response to th[e] history of disparate state laws’.).

115 H.R. Rep. No. 5056, 59th Cong., 1st Sess. 8–9 (1906) (reprinted in Hutt et al., *supra* note 62, at 429).

116
Hutt et al., *supra* note 62, at 430 (citing S. Rep. No. 74–361, 1st Sess. 2–3 1935).

117 See, eg Cohen, Donley, Rebouche, *supra* note 7.

118 21 U.S.C. § 393.

^119^

*Zogenix, Inc. v. Patrick*, No. CIV.A. 14–11,689-RWZ, 2014 WL 1454696, at ^*^2 (D. Mass. Apr. 15, 2014).

120 21 U.S.C. § 393.

121 Additionally, as discussed above, section 505–1 of the FDCA—the REMS provision—expressly requires FDA to consider burdens on the health care system and patient access in certain circumstances. 21 U.S.C. § 355–1(f)(2).

^122^
See Zettler, *Pharmaceutical Federalism*, *supra* note 22, at 872–73. Texas S.B. 4 also prohibits a ‘manufacturer’ or ‘supplier’ from providing mifepristone by mail, arguably belying an intent to regulate only the practice of medicine.

123
*Ouellette v. Mills*, 91 F. Supp. 3d 1, 9 (D. Me. 2015); *Zogenix*, 2014 WL 1454696, at ^*^2. See also Zettler, *Pharmaceutical Federalism*, *supra* note 22at 870–75 (making a similar point). *Cf. United States v. Regenerative Scis.*, LLC, 741 F.3d 1314, 1319 (D.C. Cir. 2014) (‘appellants are wrong to suggest that the scope of the FDCA depends on state-by-state definitions of the “practice of medicine”’). Field preemption is a form of implied preemption that occurs when ‘a pervasive scheme of federal regulation implicitly precludes supplementary state regulation’. CRS Federal Preemption, *supra* note 62, at 2.

^124^

*Zogenix, Inc. v. Baker*, No. 14–11,689-RWZ, 2015 WL 1206354, at ^*^2 (D. Mass. Mar. 17, 2015).

^125^

*Id.* at ^*^4.

^126^
See, eg Noah, *supra* note 13, at 53–54 (suggesting state bans on drugs that were long-ago-approved and that are premised on safety and effectiveness information that FDA did not consider may not be preempted).

127 H.B. 261 § 3. If this bill is read as a ban on the substance mifepristone, rather than solely its abortion-related uses, the bill would also ban Korlym, the mifepristone product approved for Cushing’s Syndrome.

128 The bill incorrectly states ‘Chemical abortions result in complications at a rate four times greater than the rate of complications with surgical abortions’. In fact, current scientific evidence suggests mifepristone is comparably safe to procedural abortion during the first trimester. See Nat’l Acad. of Sci., Engineering & Med., The Safety and Quality of Abortion Care in the United States 55, 56, 61, 62 (2018).

129 H.B. 261 § 2.

130 See, eg Gary Marchant, Ann Meyer, Megan Scanlon*, Integrating Social and Ethical Concerns into Regulatory Decision-Making for Emerging Technologies*, 11 Minn. J.L. Sci. & Tech. 345, 349 (2010); Patricia J. Zettler, *The FDA's Power over Non-Therapeutic Uses of Drugs and Devices*, 78 Wash. & Lee L. Rev. 379, 446 (2021).

131 See, eg U.S. Food & Drug Admin., FDA's Response to Public Comment on the Animal Cloning Risk Assessment, Risk Management Plan, and Guidance for Industry (‘The agency is not charged with addressing non-science based concerns such as the moral, religious, or ethical issues associated with animal cloning for agricultural purposes, the economic impact of products being released in commerce, or other social issues unrelated to FDA's public health mission’.), https://www.fda.gov/animal-veterinary/animal-cloning/fdas-response-public-comment-animal-cloning-risk-assessment-risk-management-plan-and-guidance (accessed November 16, 2022).

132 See, eg *Wyeth*, 555 U.S. at 564 (internal quotations and citations omitted).

133 McCuskey, *supra* note 14, at 104

134 Garland Statement, *supra* note 8 (emphasis added).

135 See, eg Robert L. Rabin, *Reflections on Tort and the Administrative State*, 61 DePaul L. Rev. 239, 249 (2012) (describing the ‘the singularity of tort in doing double duty—offering compensation as well as playing a deterrence-animated regulatory role’). See also Boyd, *supra* note at 14, 200 (2022) (‘The FDCA gives FDA the authority to regulate cosmetics, but it confers no private right of action. Tort law, by contrast, empowers people to demand a response for a wrong they have suffered’.)

136
*Mut. Pharm. Co. v. Bartlett*, 570 U.S. 472, 475 (2013). Additionally, allowing states to rely on social or moral reasons to ban FDA-approved drugs may open the door to very difficult questions about what constitutes a social or moral consideration. The line between what is a moral or social concern and a public health concern is not clearly defined, as the debate about reproductive health care itself ably demonstrates. See, eg Patricia J. Zettler, Margaret Foster Riley, Aaron S. Kesselheim, *Implementing A Public Health Perspective in FDA Drug Regulation*, 73 Food & Drug L.J. 221, 255 (2018) (‘The line between political and public health concerns is not always clear’.). *But cf.* Ed Whelan, *New York Times Botches FDA Non-Preemption of Abortion Drugs*, Nat’l Rev. (July 21, 2022).

137 FDA’s Response to Public Comment on the Animal Cloning Risk Assessment, Risk Management Plan, and Guidance for Industry, *supra* note 115.

138
*Cf. Wyeth v Levine*, 555 U.S. 555, 581 (2009) (recognizing ‘that some state-law claims might well frustrate the achievement of congressional objectives’, notwithstanding the differing purposes of state tort law and the FDCA but concluding the claims at issue in the case did not so frustrate congressional objectives). We note that these preemption arguments are less persuasive for the converse situation—states permitting sale of a drug not approved by FDA for moral, or even safety and effectiveness, reasons. See Zettler, *Pharmaceutical Federalism*, *supra* note 13, at 877–885.

139 RsMO § 188.017.4.

140 O.R.C. § 2919.195. A fetal heartbeat is typically detected around 6 weeks of pregnancy, which is roughly 2 weeks after the very earliest point a person might be able to learn that they are pregnant. At the time of writing, Ohio's law is subject to ongoing litigation. See, eg Julia Carr Smyth, *Judge Hears Arguments in Challenge to Ohio Abortion Law*, PBS (Oct. 7, 2022), https://www.pbs.org/newshour/nation/judge-hears-arguments-in-challenge-to-ohio-abortion-law.

141 There are drugs that are not approved for pregnancy termination but that may be effective for that use. Korlym, the mifepristone product approved for Cushing’s Syndrome, and misoprostol, approved for reducing the risk of NSAID (nonsteroidal anti-inflammatory drug)-induced gastric ulcers, are two examples. Complete and near-complete bans on abortion care prohibit not only the on-label use of mifepristone for abortion but also seem to prohibit the off-label abortion uses of other drugs not approved from pregnancy termination. For brief discussion of preemption challenges to state restrictions on off-label uses of Korlym specifically, see note 65, *supra*.

142 Cohen, Donley & Rebouche, *supra* note 17. See also *Food & Drug Admin. v. Brown & Williamson Tobacco Corp.*, 529 U.S. 120, 159 (2000); *West Virginia v. EPA __ U.S. __* (June 30, 2022). See also Memo in Opp. to Mot. For Leave to File Amended Compl., 12, No, 3:20-CV-652-HTW-LGI (S.D. Miss.) (arguing ‘[t]he major-questions doctrine independently dooms’ FDCA preemption arguments regarding Mississippi’s restrictions on mifepristone).

143 See Cohen, Donley & Rebouche, *supra* note 11*.*

144 See McCuskey, *supra* note 14.

145 See, eg Memo in Opp. to Mot. For Leave to File Amended Compl., 10, No, 3:20-CV-652-HTW-LGI (S.D. Miss.).

146 Whelan, *supra* note 117. Although this example presumably imagines a conflict between conservative state lawmakers and FDA determinations, it is possible to imagine similar conflicts with respect to progressive state lawmakers as well. For example, states have shown interest in regulating esthetic procedures—following a celebrity’s highly publicized 2007 death after such a procedure, California enacted a law requiring a physical examination before ‘elective facial cosmetic surgery’. See Cal. Bus. Prof. Code § 1638.2. We might imagine that a state would decide to ban health care professionals from providing any purely esthetic care, including plastic surgery that is not reconstructive, as well as esthetic uses of drugs, such as Botox for facial wrinkles, because the state believes these procedures collectively contribute to creating unrealistic beauty standards. Such a state law, like a ban on MAID should FDA ever approve a drug for that use, would have the effect of banning the sale of FDA-authorized uses of certain products within a state’s borders.

147 As noted previously in Part IV.B., in at least two cases, federal judges have rejected states’ attempts to use prescribing and dispensing regulation to regulate drugs in ways that contradict FDA regulation. *Ouellette v. Mills*, 91 F. Supp. 3d 1, 9 (D. Me. 2015); *Zogenix*, 2014 WL 1454696, at ^*^2.

148
*Mut. Pharm. Co. v. Bartlett*, 570 U.S. 472 (2013). See also *PLIVA, Inc. v. Mensing*, 564 U.S. 604 (2011).

149 But see, eg *Ulleseit v. Bayer Healthcare Pharms. Inc.*, No. 19–15778, 2021 WL 6139816, at ^*^1 (9th Cir. Dec. 29, 2021) (declining to extend the preemption analysis in *Mutual Pharmaceutical* and *Pliva* to failure-to-warn claims brought against drug distributors).

150 See, eg Hutt et.al, *supra* note 62, at 424–54. States also regulate other products—such as food and tobacco products—in ways that complement, or potentially diverge from, FDA.

151 See, eg Zettler, *Pharmaceutical Federalism*, *supra* note 13, at 875–77. We focus on examples of state laws that are more stringent than FDA requirements, because those are most analogous to mifepristone restrictions. States also enact laws or implement regulations that are more lax than FDA requirements, such as state ‘right to try’ laws that were intended to allow the distribution of unapproved drugs, or more recently, state ‘right to try 2.0’ laws focused on permitting the distribution of unapproved ‘individualized’ genetic interventions. See eg, *id.* at 881; Nicholas Florko, *Right to Try 2.0: Advocates Want to Reduce Oversight of ‘N of 1’ Therapies*, STAT (Apr. 26, 2022). Although such laws are not likely to be preempted per se—eg parties can comply with both state and federal law by complying with the more stringent federal requirements—they also are without significant legal effect, because states cannot eliminate federal obligations. See eg*,* Zettler, *Pharmaceutical Federalism*, *supra* note 13, at 877–85.

152 David & Rosenbaum, *supra* note 15, at 494.

153
*Id.* at 497. This is not to say federalism always benefits health care or public health. See, eg Abbe R. Gluck & Nicole Huberfeld, *The New Health Care Federalism on the Ground*, 15 Ind. Health L. Rev. 1, 3 (2018) (‘We can say more assuredly that the [Affordable Care Act’s] federalism served state power than we can say that its federalism produced better health policy outcomes. . ‘.); Wiley et al., *supra* note 14, at 698 (‘Health care federalism has an inconsistent and often ineffective legacy...’).

154 In forecasting these effects, we echo Hank Greely, and note that it is hard to be right when predicting the future, though we try our best here. Greely, *supra* note 6.

155 See, eg David & Rosenbaum, *supra* note 15 (describing the public health benefits of preemption defenses failing in lawsuits brought against drug manufacturers).

156 Wiggins, *supra* note 16.

157 Jo Ingles, *New Ohio Bill Would Ban Most Private Insurance Coverage Of Abortion*, WOSU (May 8, 2019), https://news.wosu.org/news/2019-05-08/new-ohio-bill-would-ban-most-private-insurance-coverage-of-abortion (accessed November 16, 2022). For brief discussion of how some of the arguments in this article would apply to contraceptive drugs and devices, see note 16, *supra*.

158 Christopher O’Donnell, *Florida Only State Not to Preorder COVID Vaccine for Young Kids*, Tampa Bay Times (June 15, 2022), https://www.tampabay.com/news/health/2022/06/15/florida-only-state-not-to-preorder-covid-vaccine-for-young-kids-report-says/ (accessed November 16, 2022).

159 See, eg Joshua Stein, *The Terrifying Prospect of a Republican War on Antidepressants*, Slate (Aug. 8, 2022), https://slate.com/technology/2022/08/republican-war-antidepressants-ssris.html (accessed November 16, 2022).

160 See, eg Patricia J. Zettler, Micah L. Berman & Efthimios Parasidis, *Drug and Vaccine Development and Access, in*Assessing Legal Responses to COVID-19 (Burris, S., de Guia, S., Gable, L., Levin, D.E., Parmet, W.E., Terry, N.P. eds., 2021).

161 The lack of uniformity may also raise a variety of challenging regulatory questions. For instance, what would the oversight of prescription drug advertising and promotion look like if drugs are federally approved but banned in some states (particularly, given that some states are seeking to criminalize speech about abortion)? See South Carolina S.B. 1373 (2021–22). See also Bess Levin, *South Carolina Republicans Basically Want to Make it Illegal to Utter the Word Abortion*, Vanity Fair (July 22, 2022) (discussing S.B. 1373). A lack of uniformity also would decrease industry incentives to engage in the expensive research needed to bring new products to market.

162 But *cf.* Zettler, Riley & Kesselheim, *supra* note 117, at 255 (discussing how political and public health concerns may be comingled). That said, it seems likely that drugs associated with stigmatized conditions—like HIV—will be particularly likely targets of state regulatory efforts. *Cf.* Doron Dorfman, *The Prep Penalty*, 63 B.C. L. Rev. 813, 815 (2022) (arguing, with respect to HIV PrEP drugs specifically, ‘decisions related to public health, on the structural and individual levels, are colored by moral judgment’).

163 See notes 139–142 and the accompanying text.

164
*Cf.* Cohen, Donley & Rebouche, *supra* note 11 at 49 (making a similar point regarding successful preemption challenges to mifepristone laws).

165 Bragg & Florence, *supra* note 84 at 278.

166 It may be that a REMS-related analysis would also extend to lawsuits involving drugs, for which no REMS has ever been required, to the extent FDA considers whether a REMS is necessary for every approval decision and for every marketed drug approved before the REMS provisions were enacted. See note 99 *supra*.

167 See, eg Nora Freeman Engstrom & Robert L. Rabin, *Pursuing Public Health Through Litigation: Lessons from Tobacco and Opioids*, 73 Stan. L. Rev. 285, 350–62 (2021).

168
*Ulleseit v. Bayer Healthcare Pharms. Inc.*, No. 19–15778, 2021 WL 6139816, at ^*^1 (9th Cir. Dec. 29, 2021). But *cf.* Stephen McConnell, *Ninth Circuit Is Gonna be the Ninth Circuit: Preemption, Fraudulent Joinder, and Lassitude*, Drug & Device Law (July 13, 2022) (strongly critiquing the *Ulseseit* opinion), https://www.druganddevicelawblog.com/2022/07/ninth-circuit-is-gonna-be-the-ninth-circuit-preemption-fraudulent-joinder-and-lassitude.html (accessed November 16, 2022).

169 U.S. Food & Drug Admin., Generic Drugs, https://www.fda.gov/drugs/buying-using-medicine-safely/generic-drugs#:~:text=In%20the%20United%20States%2C%209,filled%20are%20for%20generic%20drugs./ (accessed November 16, 2022).

170
*Cf. Food & Drug Admin. v. Am. Coll. of Obstetricians & Gynecologists*, 141 S. Ct. 578, 579 (2021) (Sotomayor, J., dissenting) (comparing mifepristone to opioids, noting ‘[g]overnment policy now permits patients to receive prescriptions for powerful opioids without leaving home, yet still requires women to travel to a doctor's office to pick up mifepristone, only to turn around, go home, and ingest it without supervision’.); Noah, *State Regulatory Responses*, *supra* note 66, at 667 n.36 (2020) (suggesting state opioid restrictions may be vulnerable to conflict preemption challenges because opioids are subject to REMS).

171 FDA has required a REMS for extended-release/long-acting opioid analgesics since 2012 and for immediate release opioids since 2018. See U.S. Food & Drug Admin, Opioid Analgesic REMS, What Updates Have Been Made to the REMS?, https://www.accessdata.fda.gov/scripts/cder/rems/index.cfm?event=RemsDetails.page&REMS=17 (accessed November 16, 2022).

172 O.A.C. § 4731-11-14(D).

173 U.S. Food & Drug Admin., Opioid Patient-Prescriber Agreement, https://www.fda.gov/drugs/safe-use-initiative/safe-use-initiative-current-projects#opioidppa (accessed November 16, 2022).

174
*Cf. Zogenix, Inc. v. Baker*, No. 14–11689-RWZ, 2015 WL 1206354, at ^*^4 (D. Mass. Mar. 17, 2015) (noting that obstacle preemption challenges may be successful when state regulation falling short of a ban nevertheless limits availability of a drug).

175 See, eg William Fleischman et al., *Association of a Risk Evaluation and Mitigation Strategy Program With Transmucosal Fentanyl Prescribing*, 2 JAMA Network Open e191340 (2019); Ameet Sarpatwari & Gregory Curfman, *Mitigating Health Risks of Prescription Drugs: Lessons From FDA Oversight of Opioid Products*, 321 J. Am. Med. Ass’n 651 (2019); Ameet Sarpatwari et al., *Changes in Erythropoiesis Stimulating Agent Use Under a Risk Evaluation and Mitigation Strategy (REMS) Program*, 44 Drug Safety 327 (2021). See also Lars Noah, *Federal Regulatory Responses to the Prescription Opioid Crisis: Too Little, Too Late?,* 2019 Utah L. Rev. 757, 781 (2019) (critiquing FDA’s use of its REMS authority for opioids).

176
Dep’t of Health & Hum. Servs, Office of Inspector General, FDA Lacks Comprehensive Data To Determine Whether Risk Evaluation and Mitigation Strategies Improve Drug Safety (Feb. 2013), https://oig.hhs.gov/oei/reports/OEI-04-11-00510.asp; Dep’t of Health & Hum. Servs, Office of Inspector General, FDA’s Risk Evaluation and Mitigation Strategies: Uncertain Effectiveness in Addressing the Opioid Crisis (Sept. 2020), https://oig.hhs.gov/oei/reports/OEI-01-17-00510.asp (accessed November 16, 2022).

177
*Cf.* Wiley et al., *supra* note 14, at 701 (discussing the COVID-19 pandemic, explaining ‘federalism has sowed dysfunction in testing, treatment, and vaccination policy’).

178 See, eg 2022 ACOG Letter, *supra* note 5. We do not intend to imply that there are no ob/gyn physicians or ob/gyn professional organizations that are anti-abortion. Our point is simply that there is general agreement among experts, and similar conclusions coming from credible studies, about what is necessary for safe and effective use of mifepristone. By contrast, what constitutes appropriate regulation of opioid analgesics is a much more complicated question.

179 See, eg George Comerci, Jr et al., *Controlling the Swing of the Opioid Pendulum*, 378 N. Engl. J. Med. 691 (2018); Kelly K. Dineen, *Addressing Prescription Opioid Abuse Concerns in Context: Synchronizing Policy Solutions to Multiple Complex Public Health Problems*, 40 L. & Psychol. Rev. 1 (2016); Daniel S. Goldberg & Ben Rich, *Pharmacovigilence and the Plight of Chronic Pain Patients: In Pursuit of a Realistic and Responsible Ethic of Care*, 11 Ind. Health L. Rev. 83 (2014); Ellen Meara et al., *State Legal Restrictions and Prescription-Opioid Use among Disabled Adults*, 375 N. Engl. J. Med. 44 (2016).

180 To return to the opioid treatment agreement example, as previously noted, FDA recommends use of an opioid treatment agreement, and CDC, likewise, recommends use. See Tuesday M. MacAuliff Staehler & Laura C. Palombi, *Beneficial Opioid Management Strategies: A Review of the Evidence for the Use of Opioid Treatment Agreements*, 41 Substance Abuse 208 (2020). See also Joshua B. Rager & Peter H. Schwartz, *Defending Opioid Treatment Agreements: Disclosure, Not Promises*, 47 Hastings Center Rep. 24, 25 (2017) (describing the ethical basis for the use of opioid treatment agreements as ‘not to justify the system of monitoring and supervision but instead to disclose its requirements to patients’). *Cf.* Jose M. Fernandez & Jayani Jayawardhana, *The Effect of Pill Mill Legislation on Suicides*, Health Servs. Res. (published online 2022; forthcoming in print) (reporting study results ‘indicat[ing] that pill mill legislation has been effective in reducing total suicides among females in the age group 45–64, and drug-related suicides in the population’).

181 Zettler, *Pharmaceutical Federalism*, *supra* note 13, at 895–900.

182 Zogenix, Inc. v. Patrick, 2014 WL 1454696, at ^*^1 (D. Mass. Apr. 15, 2014).

183 See, eg Zettler, *Pharmaceutical Federalism*, *supra* note 13, at 897.

184 See *id.* There are other examples of federal pharmaceutical policy following patterns developed at the state level, even when state regulation is not enforced, is preempted, or is without significant effect because it attempts to establish requirements more permissive than FDAs. These include the federal requirement for a track and trace system for the drug supply chain enacted in the Drug Quality and Security Act of 2013, which followed several state efforts to require such a system, as well as the federal Right to Try Act of 2018, creating a new pathway for drug manufacturers to provide non-trial preapproval access to investigation drugs, which came after over 40 states passed similar laws. See *id.*

185 See, eg David S. Cohen, Greer Donley & Rachel Rebouche, *Re-Thinking Strategy After Roe*, 75 Stanford L. Rev. Online 1 (2022).

186
*Cf.* Lewis A. Grossman, *The Origins of American Health Libertarianism*, 13 Yale J. Health Pol’y, L. & Ethics 76 (2013) (‘Modern American history has given rise to movements for access to abortion, life-ending drugs, unapproved cancer treatments, and medical marijuana’.); Seema Shah & Patricia J. Zettler, *From A Constitutional Right to A Policy of Exceptions: Abigail Alliance and the Future of Access to Experimental Therapy*, 10 Yale J. Health Pol’y, L. & Ethics 135, 146–49 (2010) (discussing arguments regarding medical self-defense, abortion, and FDA’s approval authority for drugs).

